# HLA Epitopes: The Targets of Monoclonal and Alloantibodies Defined

**DOI:** 10.1155/2017/3406230

**Published:** 2017-05-24

**Authors:** Nadim El-Awar, Vadim Jucaud, Anh Nguyen

**Affiliations:** Terasaki Foundation Laboratory, Los Angeles, CA, USA

## Abstract

Sensitization to human leukocyte antigens (HLA) in organ transplant patients causes graft rejection, according to the humoral theory of transplantation. Sensitization is almost ubiquitous as anti-HLA antibodies are found in almost all sera of transplant recipients. Advances in testing assays and amino acid sequencing of HLA along with computer software contributed further to the understanding of antibody-antigen reactivity. It is commonly understood that antibodies bind to HLA antigens. With current knowledge of epitopes, it is more accurate to describe that antibodies bind to their target epitopes on the surface of HLA molecular chains. Epitopes are present on a single HLA (private epitope) or shared by multiple antigens (public epitope). The phenomenon of cross-reactivity in HLA testing, often explained as cross-reactive groups (CREGs) of antigens with antibody, can be clearly explained now by public epitopes. Since 2006, we defined and reported 194 HLA class I unique epitopes, including 56 cryptic epitopes on dissociated HLA class I heavy chains, 83 HLA class II epitopes, 60 epitopes on HLA-DRB1, 15 epitopes on HLA-DQB1, 3 epitopes on HLA-DQA1, 5 epitopes on HLA-DPB1, and 7 MICA epitopes. In this paper, we provide a summary of our findings.

## 1. Introduction

Sensitization to HLA antigens in organ transplant patients causes graft rejection, according to the humoral theory of transplantation [1]. Sensitization is almost ubiquitous as it is evident in the detection of anti-HLA antibodies in the sera of recipients—in one study, almost all patients waiting for regraft of a kidney transplant have anti-HLA antibodies [2]. Determining specificity of the anti-HLA antibody has advanced in recent years using recombinant HLA single antigens (SA) coated on color-coded Luminex beads [3]. The reactivity of anti-HLA antibodies with HLA antigens and the phenomenon of cross-reactivity has been the subject of investigation for decades. Amino acid sequences of the HLA molecules which greatly contributed to our understanding of antibody and antigen reactivity has been introduced since 1963 [4–12]. Antibodies are commonly described as binding to HLA antigens; however, it is more accurate to describe the reactivity of the antibody as binding to specific epitopes on the surface of HLA antigens—epitopes are conformational amino acid arrangements and are the targets of antibodies. Some epitopes are private, found exclusively on one antigen; others are public epitopes shared by two or more antigens. The phenomenon of cross-reactivity in HLA testing, often explained as cross-reactive groups (CREGs), of antigens with antibody can be clearly explained now by public epitopes—an antibody targeting a public epitope shows positive reaction with all antigens sharing the epitope.

Since 2006, we defined and reported on 194 HLA class I unique epitopes, including 138 epitopes on intact HLA class I (heavy chain + *β*2m + peptide), and 56 cryptic epitopes on dissociated HLA class I (heavy chain only) [13–18]. 110 epitopes on intact HLA class I were defined using murine monoclonal and human alloantibodies, and the remaining 28 epitopes were defined with naturally occurring anti-HLA antibodies. Naturally occurring (natural) HLA antibodies found in cord blood and healthy males were used to define the 56 cryptic epitopes on dissociated HLA class I. In addition, 83 HLA class II unique epitopes were defined and reported, including 60 epitopes on HLA-DRB1, 15 epitopes on HLA-DQB1, 3 epitopes on HLA-DQA1, and 5 epitopes on HLA-DPB1 [15, 19–22]. All HLA-DRB1 epitopes were defined using solely amino acid sequence data, in contrast to HLA-DQA1, HLA-DQB1, and HLA-DPB1 epitopes that were defined using human alloantibodies. Lastly, we defined and reported on 7 MICA epitopes using human alloantibodies [15, 22]. In this paper, we provide a summary of our findings.

## 2. Materials and Methods

**Figure 1:**
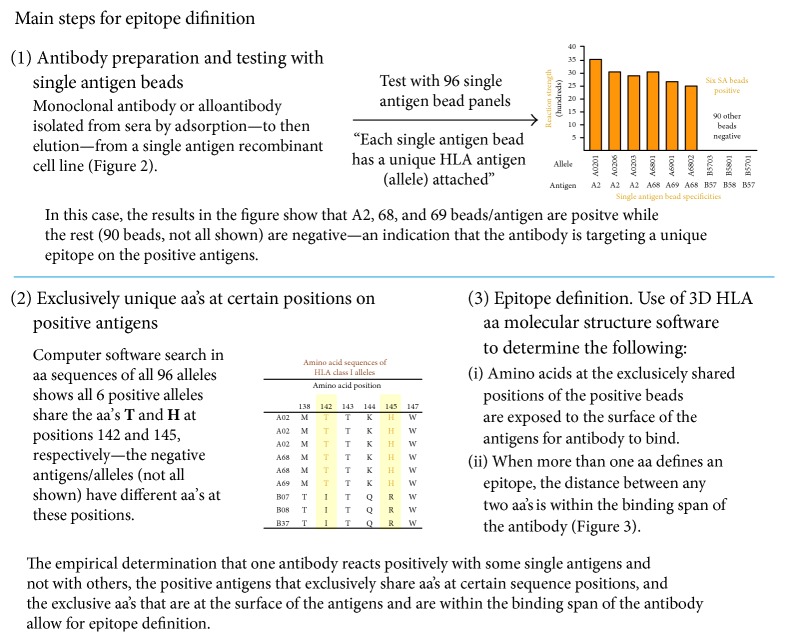
Main empirical testing steps to define HLA epitopes.

The principle we used to define HLA epitopes is summarized in (Figure 1). Briefly, if an antibody is determined to test positive with certain HLA antigens and negative with others, it is reasonable to assume that the antibody is targeting a specific epitope on the positive antigens. Epitopes are conformational arrangements of amino acids (aa) at sequence positions on the surface of antigens that must be within the binding span of the antibody. To define an epitope, a computer search, in published aa sequences of tested antigens, was performed to identify exclusively shared aa at one or more sequence positions among the positive antigens—these amino acids define the epitope.

**Figure 2:**
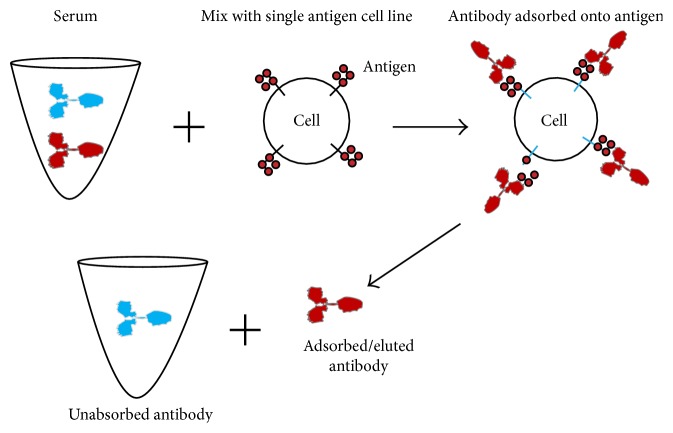
Alloantibody adsorption/elution with recombinant single antigen cell line. The antibody is eluted with an acidic buffer, and the eluate is neutralized with TRIS buffer.

Murine monoclonal antibodies or transplant recipient and healthy male HLA antibodies isolated from sera and cord blood by first adsorbing them onto appropriate recombinant HLA (rHLA) single antigen cells, then eluted with an acidic buffer (ImmunoPure IgG elution buffer, Pierce, Rockford, IL), and neutralized with 1 M TRIS-HCl pH 9.5 (Figure 2()) were all tested with the single antigen beads (One Lambda Inc., Canoga Park, CA) to determine the specificity of the antibodies [14]. HLA class I SA beads treated with a buffer that dissociates the peptide and the beta-2-microglobulin (*β*2m) from the heavy chain of the intact HLA antigens on the beads [17] were used to reveal the specificity of antibodies targeting epitopes on dissociated class I heavy chains. MFI values of 1000 or above were considered positive except when the overall reactions of an eluted antibody were weak, a cutoff of MFI 400 was used.

**Figure 3:**
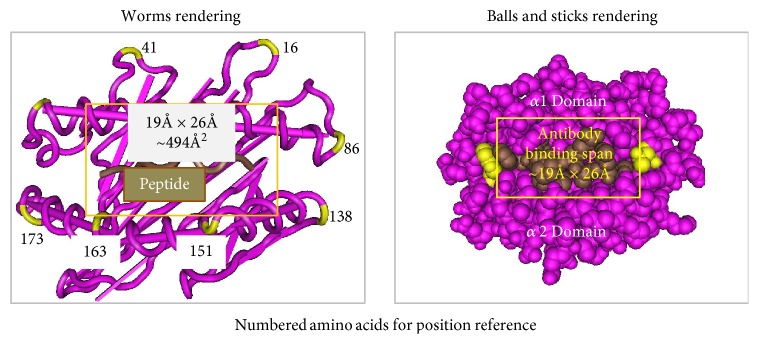
Top view of HLA class I heavy chain 1 and 2 domains. Rectangles show approximate binding span area of antibody.

Computer software was utilized to search for exclusive amino acids in the structure of antigens showing positive reactions with an antibody. Searches were performed within sequences of HLA class I heavy chains, MICA antigens, DR beta chains, DQ beta and alpha chains, and DPB chains. All amino acid sequences were obtained from the HLA Informatics Group at the Anthony Nolan website [23]. One or more amino acids found exclusively at the same sequence positions in the chains of positive antigens, but not in the sequence positions of negative antigens, were designated as the defining amino acids for an epitope. The defining amino acid(s) had to be within the antibody binding span [24, 25] — estimated at 494 Å2–750 Å2 area (Figure 3()) and the aa(s) must be exposed at the surface of the antigen—exceptions are noted between parentheses (Table 1).

**Figure 4:**
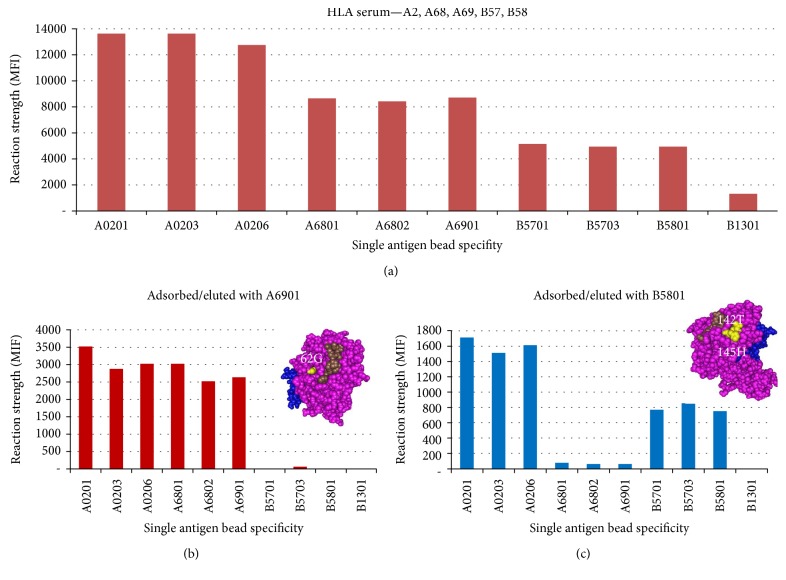
Alloserum with specificity A2, A68, A69, B57, and B58 (a). Antibody eluted from adsorption with A6901 recombinant cells has the specificity of A2, A68, and A69 (b). Antibody eluted from adsorption with B5801 recombinant cells has the specificity of A2, B57, and 58 (c).

The efficacy of isolating HLA antibody from HLA sera with adsorption and elution assays, testing the eluted antibody with the SA beads to determine specificity and the definition of the epitope on the surface of positive antigens (corresponding to antibody specificity) are shown in (Figure 4). Alloserum with determined specificity A2, A68, A69, B57, and B58 was adsorbed separately with SA rHLA A6901 and B5801 cells. Eluted antibodies tested with the SA beads showed specificity A2, A68, and A69 and A2, B57, and B58, respectively. HLA antigens A2, A68, and A69 share an epitope defined by glycine (G) at position 62; therefore, 62G defines the epitope. Similarly, HLA antigens A2, B57, and B58 share an epitope defined by threonine (T) at position 142 or histidine (H) at position 145; therefore 142T or 145H define the epitope.

## 3. Results

### 3.1. Class I Epitopes on Intact Antigens

138 unique epitopes were defined for one or a group of two or more intact HLA class I antigens. 110 unique epitopes were defined by using SA beads (Table 1, partial list; complete table in the supplemental information available online at https://doi.org/10.1155/2017/3406230) assays to test eluted alloantibodies that were adsorbed from human sera onto the surface of mammalian rHLA single antigen cells then eluted, and murine monoclonal antibodies to determine specificity of each antibody. Epitopes were defined by identifying exclusively unique amino acids among the positive antigens. Also defined were 28 unique epitopes targeted by naturally occurring anti-HLA antibodies found in sera of healthy males and in cord blood (Table 2). All epitopes were defined by identifying exclusively unique amino acids among the positive antigens. Here, we present partial lists in tables and example figures of epitopes—complete tables and other figures can be found in the supplemental information document.

The number of epitopes defined for each antigen, using human alloantibodies, varied from 4 to 23 (Table 3). In general, there was no correlation between the number of epitopes and the frequency of antigen in the population. For example, for HLA A2, the most frequent antigen (*f* = 30.3% to 54%), we defined 16 epitopes while for A25, with a frequency of *f* = 0.0% to 6.1%, we defined 19 epitopes. Class I epitopes were found to be shared by antigens of the same locus or by inter-locus antigens—BC or ABC. Epitopes are defined by 1, 2, 3, or 4 aa's. For example, 17 A-locus epitopes were defined by 1 aa, two B-locus epitopes by 4 aa's, or two ABC loci epitopes defined by 2 aa's. In addition, amino acids and positions on the HLA class I heavy chain epitopes were found at varying frequencies in epitope definitions. The most frequent was position 163 located in the alpha 2 domain, and the aa threonine (T) was found to be the most frequent in our studies.

The following examples illustrate HLA class I epitopes for the A-locus, B-locus, and C-locus and AB-, BC-, and ABC-loci antigens. Illustration shows SA beads specificity, antigens sharing the epitopes, and their position on the HLA class I heavy chain.

**Figure 5:**
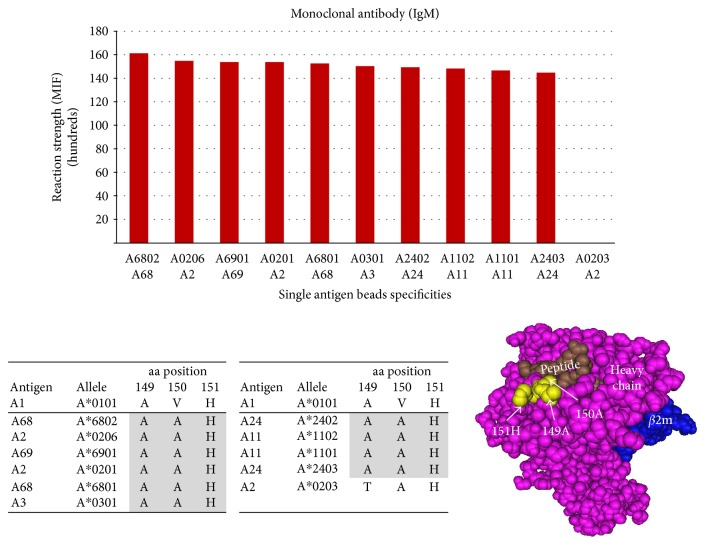
Epitope 422 shared by A-locus antigens A2, A3, A11, A24, A68, and A69 defined by the aa acid combination 149A + 150A + 151H. One amino acid substitution at position 149 (aa T substituted for aa a) could be the reason that A2 allele A^∗^0203 is negative while alleles HLA A^∗^0201 and A^∗^0206 are positive.

A-locus: Epitope 422 is shared by A-locus antigens A2, A3, A11, A24, A68, and A69 defined by 149A + 150A + 151H combined. Three amino acids at three positions are necessary to define this epitope; indeed, HLA − A^∗^02 : 01 and A^∗^02 : 06 are positive while A^∗^02 : 03 is negative. A^∗^02 : 01 and A^∗^02 : 06 share epitope 422 defined as 149A + 150A + 151H, while negative antigen A^∗^02 : 03 does not share the epitope. A^∗^02 : 03 has 150A + 151H but has threonine (T) at position 149 instead of alanine (A)—one amino acid difference in the epitope renders the antibody to be nonreactive with A^∗^02 : 03. The aa defining epitope 422 are exposed at the surface of the heavy chain and are within the binding span of the HLA antibody. The furthest amino acids are 7.88 Å apart (Figure 5).

**Figure 6:**
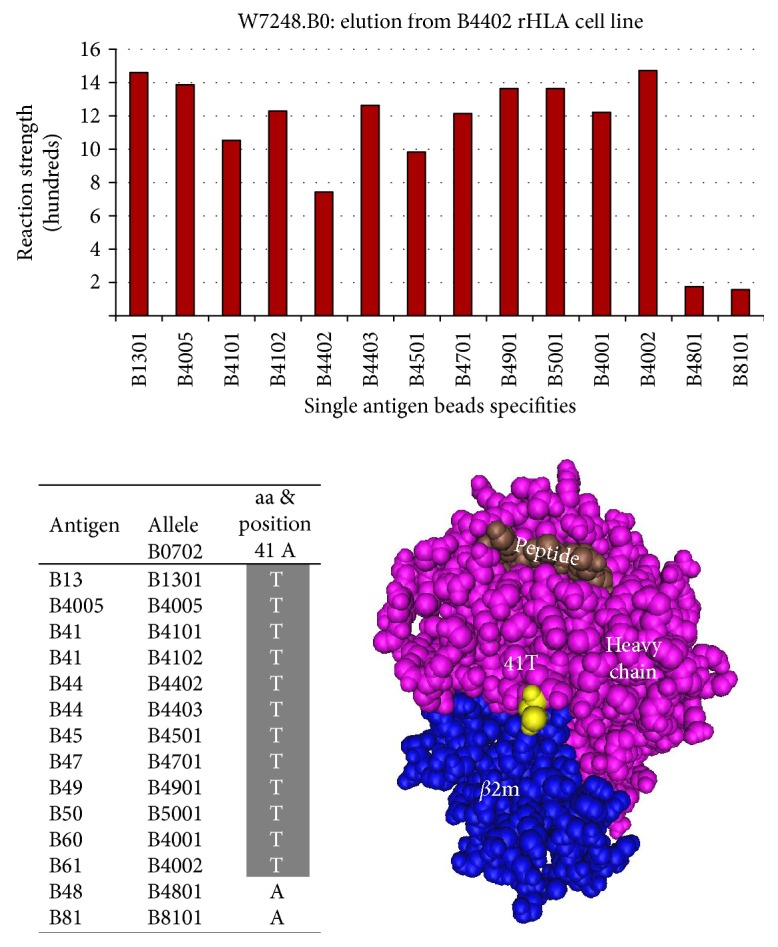
Epitope 21 shared by the B-locus antigens B13, B4005, B41, B44, B45, B47, B49, B50, B60, and B61 and defined by 41T.

B-locus: Epitope 21 is shared by B-locus antigens B13, B4005, B41, B44, B45, B47, B49, B50, B60 (B4001), and B61 (B4002) and defined by 41T. Threonine (T) is exclusively unique to the antigens at position 41 located in the alpha 1 domain of the HLA class I heavy chain (Figure 6).

**Figure 7:**
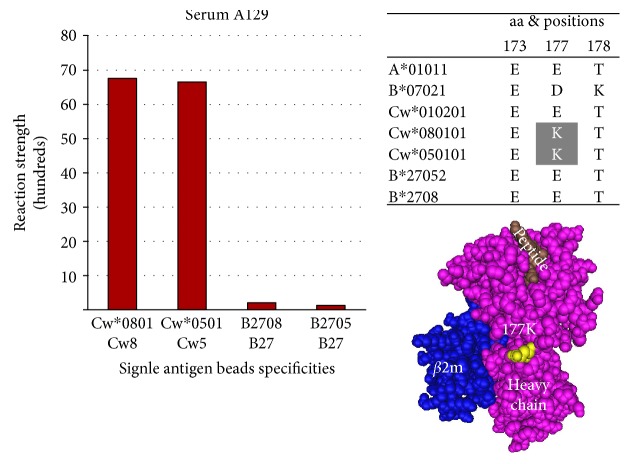
Epitope 40 shared by the C-locus antigens Cw^∗^0801 and Cw^∗^0501 and defined by 177K.

C-locus: Epitope 40 shared by the C-locus antigens Cw^∗^0801 and Cw^∗^0501 and defined by 177K located in the alpha 2 domain of the HLA heavy chain (Figure 7).

AB-Loci: Epitope 205 shared by the AB-loci A32, A74, B7, B8, B4005, B41, B42, B48, B60, B61, B73, and B81 and defined by 109L + 131R—the two positions are 11.8 Å apart and therefore within the binding span of the antibody. Also, the C-locus antigens Cw1, Cw2, Cw4, Cw5, Cw6, Cw7, Cw8, Cw9, Cw10, Cw12, Cw14, Cw15, Cw16, Cw17, and Cw18 share the same amino acids at positions 109 and131 but were not tested with the C-locus beads at the time of the study.

Epitope 24 is shared by the AB-loci Bw4-associated antigens A23, A24, A25, A32, B13, B2705, B37, B38, B44, B47, B49, B51, B52, B53, B57, B58, B59, B63, and B77 and defined by either 82L or 83R located in the alpha 1 domain of the HLA class I heavy chain.

Epitope 423 is shared by the AB-loci Bw4-associated antigens A23, A25, A32, B2705, B37, B38, B44, B47, B49, B51, B52, B53, B57, B58, B59, B63, and B77 (A24, B13 negative) and defined by 83R + 144Q + 145R. This epitope was defined using a monoclonal antibody and seems to be a variant of epitope 24 shared by all Bw4-associated antigens. Other variants of the BW4-associated antigens epitope (epitopes 249 and 250) also defined with monoclonal antibodies (Table 1).

BC-Loci: Epitope 246 is shared by BC-loci antigens B46, B73, Cw1, Cw7, Cw8, Cw9, Cw10, Cw12, Cw14, and Cw16 and defined by 76V + 80N. The two amino acids are 8.69 Å apart which is within the binding span of the HLA antibody.

**Figure 8:**
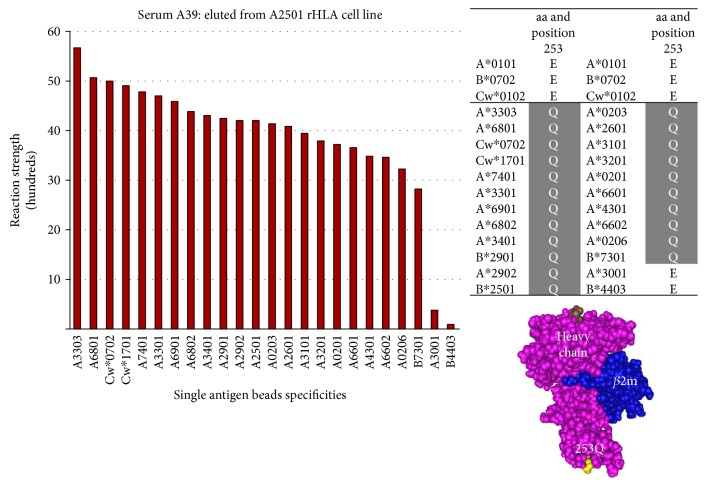
Epitope 38 shared by the ABC-loci antigens A2, A25, A26, A29, A31, A32, A33, A34, A43, A66, A68, A69, A74, B73, Cw7, and Cw17 and defined by the amino acid glutamine (Q) at position 253.

ABC-loci: Epitope 38 is shared by the ABC-loci antigens A2, A25, A26, A29, A31, A32, A33, A34, A43, A66, A68, A69, A74, B73, Cw7, and Cw17 and defined by the amino acid glutamine (Q) at position 253 of the HLA class I heavy chain located in the alpha 3 domain proximal to the cell membrane (Figure 8).

### 3.2. Cryptic Epitopes on Dissociated HLA Class I Antigens

Naturally occurring anti HLA antibodies were detected in nonalloimmunized healthy males [26], and 96 of their target epitopes were defined [16]. 58 natural antibodies are only reactive with dissociated HLA class I antigens, heavy chain only (Table 4). 56 unique epitopes on dissociated HLA class I defined [16].

**Figure 9:**
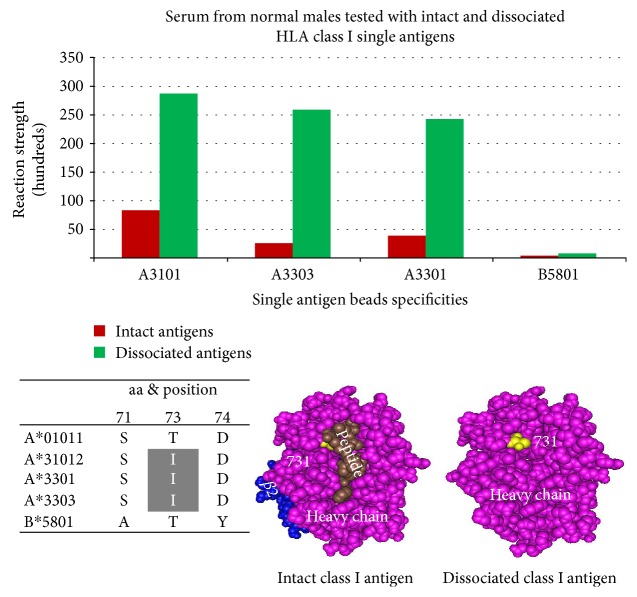
Epitope 5007 shared by the HLA class I A-locus antigens A31 and A33 and defined by isolucine (I) at position 73. The epitope is accessible on the dissociated antigens and show stronger reactivity when the peptide has been dissociated from the heavy chain. Position 73 is not exposed in an intact HLA class I antigen. After acid buffer treatment and neutralization of the eluate, epitope 5007 becomes exposed and reacts with the antibody 10-fold.

Epitope 5007 is shared by the HLA class I A-locus antigens A31 and A33 and defined by isoleucine (I) at the cryptic position 73. Antibody reactivity with the intact antigen is obstructed because position 73 is located under the peptide. It is slightly reactive with the intact HLA class I antigens. Reactivity increased by up to 10-fold with the dissociated antigens (heavy chain only)—when *β*2m and the peptide are dissociated from the heavy chain (Figure 9).

Epitope 5024 is shared by the HLA class I B-locus antigens B7, B42, B54, B55, B56, B67, B81, and B82 and defined by 66I + 70Q. Reactions strength of the antibody is stronger with the unobstructed epitope after dissociation of the peptide from the heavy chain.

Epitope 5037 is shared by the HLA C-locus antigens Cw4, Cw6, Cw17, and Cw18 and defined by 73A + 77N. Antibody reaction strength increases with the unobstructed epitope after removal of the peptide.

### 3.3. MICA Epitopes

MICA or MHC class I polypeptide-related sequence A antigens have similar aa structure as the HLA class I ABC heavy chains. However, MICA antigens are not associated with a peptide and beta 2 microglobulin. Seven epitopes were defined for MICA antigens (Table 5).

**Figure 10:**
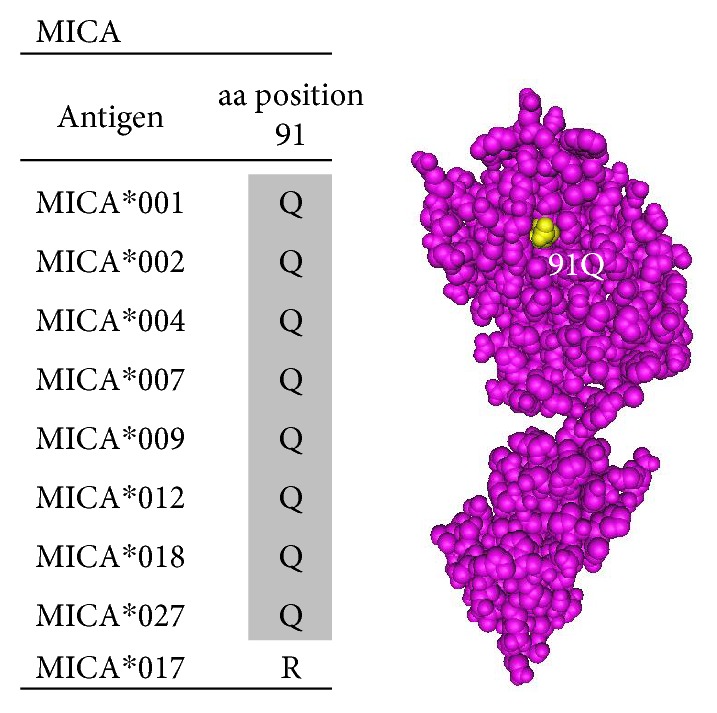
Epitope 6002 shared by MICA antigens MICA^∗^001, 002, 004, 007, 009, 012, 018, and 027 and defined by glutamine (Q) at position 91.

Epitope 6002 is shared by MICA antigens MICA^∗^001, 002, 004, 007, 009, 012, 018, and 027 and defined by glutamine (Q) at position 91; therefore, 91Q defines the epitope (Figure 10).

Epitope 6004 is shared by MICA antigens MICA^∗^04, 009, and 027 and defined by (36Y), 129V, or 173E.

### 3.4. Class II Epitopes

#### 3.4.1. HLA-DRB1 Epitopes

Unlike class I epitopes, the 60 HLA class II B1 epitopes were defined based solely on amino acid sequence of the DR antigen beta chain where all epitopes can be defined by one single amino acid at one position (Table 6). The number of epitopes for each DR antigen was from 8 to 21 epitopes.

**Figure 11:**
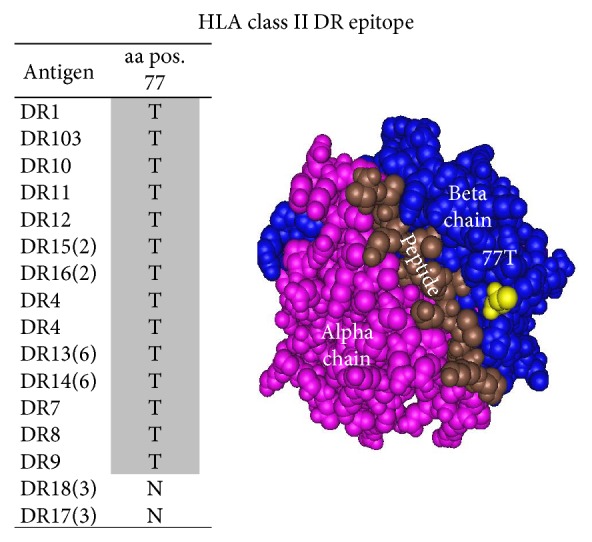
Epitope 1028 shared by class II DR antigens DR1, DR4, DR7, DR9, DR10, DR11, DR12, DR13, DR14, DR15, DR16, DR51, DR53, and DR103 and defined by threonine (T) at position 77.

For example, epitope 1028 is shared by class II DR antigens DR1, DR4, DR7, DR9, DR10, DR11, DR12, DR13, DR14, DR15, DR16, DR51, DR53, and DR103 and defined by threonine (T) at position 77 (Figure 11).

#### 3.4.2. HLA-DQA1 and HLA-DQB1 Epitopes

Eighteen HLA class II DQB1 and DQA1 epitopes are defined using the adsorption and elution assays described in the Materials and Methods above. Fifteen of the epitopes are located on the beta chain of the DQ antigen and three on the alpha chain (Table 7). The number of epitopes for DQB chains was 4–8 and only one for DQA chains (Table 8).

**Figure 12:**
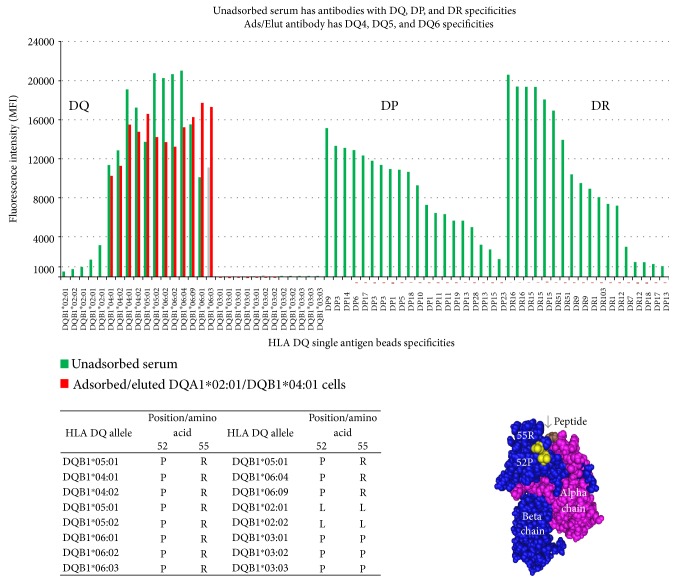
Unabsorbed serum has antibodies with specificity to DR, DQ, and DP antigens (green bars). After adsorbing the serum with rHLA DQ cells, the eluted antibody shows specificity to DQ antigens only (red bars).

Sera from allosensitized patients can be expected to have anti-HLA antibodies to class I and II antigens. As illustrated in (Figure 12), this serum has antibodies directed against DR, DQ, and DP antigens. The serum was adsorbed with DQA1^∗^02 : 01/DQB1^∗^04 : 01 rHLA cells and the eluted antibody reacted with DQ4, DQ5, and DQ6 antigens which share epitope 2007 (Table 7).

One antigen mismatch can elicit an immune response to several epitopes on an HLA antigen. A serum from renal transplant patient with DQA1^∗^02 : 01/DQB1^∗^02 : 02 mismatch has two antibodies. One antibody targets epitope 2017 (defined by histidine (H) in position 52) on the DQA1^∗^02 : 01 alpha chain and the other antibody targets epitope 2001 (defined by leucine (L) in position 52) on the DQB1^∗^02 : 02 beta chain (Table 7). As shown in the table, alternative epitope definitions are separated by “/.”

**Figure 13:**
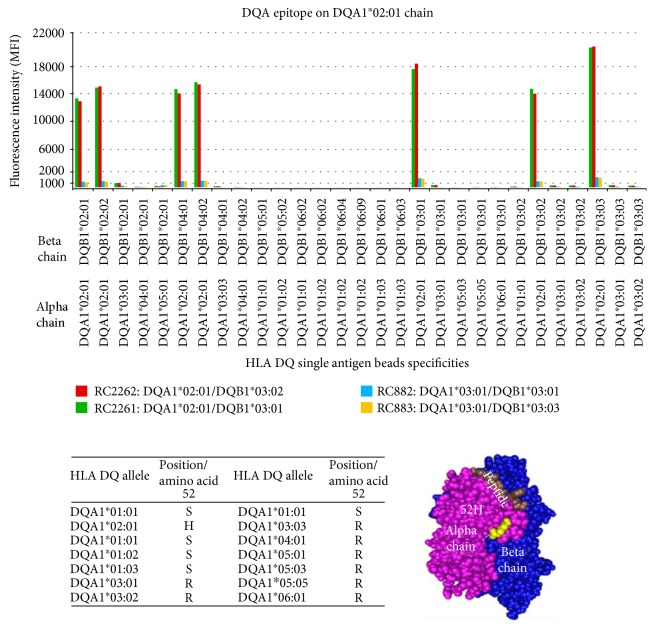
Several DQ antigens (heterodimers) with the DQA1^∗^02 : 01 chain are shown below. They all share epitope 2017 which is defined by histidine (H) in position 52 of DQA1^∗^02 : 01 chain. Eluted antibodies from relevant DQ antigens are positive (red and green bars). Eluted antibodies from irrelevant (no DQA1^∗^02 : 01) are negative (yellow and blue bars).

The efficacy of adsorption and elution assays is demonstrated where one serum with DQ specificity, including DQA1^∗^02 : 01, underwent four separate adsorptions and elutions with rHLA DQ cells. Two of the cells have the relevant DQA1^∗^02 : 01 chain, and the eluted antibodies show positive reactions with all heterodimers that contain the DQA1^∗^02 : 01chain (red and green bars). However, eluents from adsorptions with irrelevant cells (no DQA1^∗^02 : 01 chain) showed negative reactions (yellow and blue bars) (Figure 13).

The following examples illustrate HLA-DQA1 and HLA-DQB1 epitopes. Illustration shows SA beads specificity, antigens sharing the epitopes and their position on the DQA1 and DQB1 chains.

HLA-DQA1 epitopes: epitope 2018 is shared by the alpha chains of the DQ4, DQ5, and DQ6 antigens and defined by glutamine (Q) at position 53.

HLA-DQB1 epitopes: epitope 2002 is shared exclusively by the beta chains of the DQ4 antigen and defined by leucine (L) in position 56.

Epitope 2010 is shared by the beta chains of DQ antigens DQ4, DQ5, DQ6, DQ8, and DQ9 and defined by 45G + 46V.

Epitope 2022 is exclusive to DQB1^∗^05 : 01 chain on the DQ5 antigen and defined by 125S + 126Q.

Epitope 2006 is shared by DQB1^∗^03 : 01 (DQ7), DQB1^∗^03 : 02 (DQ8), and DQB1^∗^03 : 03 (DQ9) and defined by proline (P) at position 55 on the beta chains of the DQ antigens.

#### 3.4.3. HLA-DPB1 Epitopes

Five HLA class II DPB epitopes were defined. Four of the epitopes required 3-4 amino acids for definition, and one was defined by a single amino acid/position (Table 9).

Epitope 4001 is shared by the HLA class II DPB1 chains of the DP antigens DPB1^∗^01 : 01, DPB1^∗^03 : 01, DPB1^∗^05 : 01, DPB1^∗^09 : 01, DPB1^∗^10 : 01, DPB1^∗^11 : 01, DPB1^∗^13 : 01, DPB1^∗^14 : 01, DPB1^∗^17 : 01, and DPB1^∗^19 : 01 and defined by 84D + 85E + 86A + 87V. All four amino acids needed to define the epitope. Negative antigens that did not share epitope 4001 are shown as gray bars (Figure 4()).

**Figure 15:**
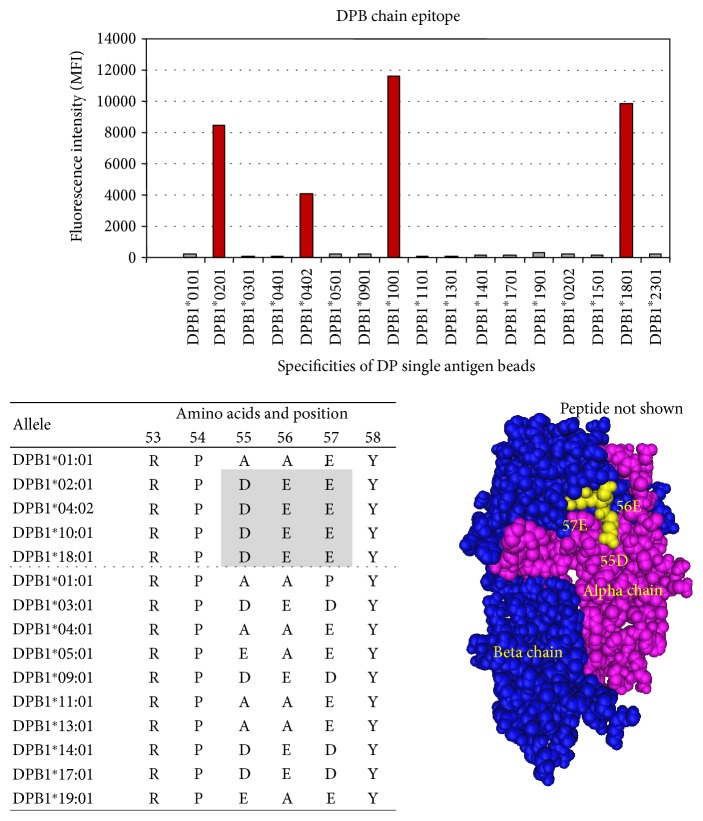
HLA class II DP epitope 4003 shared by DPB chains DPB1^∗^0201, DPB1^∗^0402, DPB1^∗^1001, and DPB1^∗^1801 (red bars) and defined by 84D + 85E + 86A + 87V. Negative antigens that did not share epitope 4003 are shown in (gray bars).

HLA class II DP epitope 4003 is shared by DPB1 chains DPB1^∗^02 : 01, DPB1^∗^04 : 02, DPB1^∗^10 : 01, and DPB1^∗^18 : 01 (red bars) and defined by 84D + 85E + 86A + 87V. Negative antigens that did not share epitope 4003 are shown in gray bars (Figure 15).

## 4. Discussion

Cross-reactivity of antibodies with HLA antigens has been investigated for decades [4, 5, 7–9, 27]. Studies to identify HLA epitope, the target of antibodies, started more than 50 years ago [10], and numerous other studies followed since then [5, 6, 11, 28–34]. The amino acid structure of HLA antigen chains was reported for the HLA A2 in 1987 [12], and now, complete sequences of all known HLA antigen chains are readily available online [23]. Single antigens expressed on a mammalian cell line allowed us to simplify adsorption/elution assays and isolate one antibody from multispecific allosera, with multiple antibodies, and test the isolated antibody with the single antigen beads to more accurately determine antibody specificity. Isolated antibodies tested with large panels of HLA class I or class II single antigen beads were shown to be positive with certain antigens of the bead panels and negative with others. It is, therefore, reasonable to assume that the positive antigens share a public epitope which can easily be confirmed by looking at the amino acid sequences of these antigens.

HLA single antigen bead assays are simplified assigning anti-HLA antibody specificities by simply listing all antigens that are positive with the serum or antibody. Because a positive antibody-antigen reaction indicates binding of antibody to the single antigen on the bead, we postulate that the antibody must be specific to the antigen. However, the single antigen beads assay often reveals more antibody specificities than other antibody detection assays. This is clearly seen when an immunological response to a mismatched antigen produces antibody specificity to nondonor antigens and in some cases unexpectedly to rare antigens. HLA antigens share public epitopes; therefore, the extra antibody specificity of non-donor-specific and rare antigens can now be explained as antibody binding to public epitopes located on the positive antigens. Defining epitopes of HLA gives us better understanding of the breadth of non-donor-specific specificities found in sera. For example, specificity of antibodies to rare antigens like A80 and B76 were unexpectedly higher than the antigens' frequency (<0.5%) in the general population. The two antigens have 9 and 13 epitopes, respectively (Table 3).

HLA epitopes were defined using computer software by searching, in published sequences of class I and class II antigens, for exclusive amino acids at the same position(s) that are shared by all positive-reacting antigens. Amino acid sequences and the 3D structures of available HLA antigens, used to ensure that aa's are exposed on the surface of the antigens, helped in defining close to 300 epitopes. Assay-positive antigens that share epitopes, defined by exclusively shared aa's, correspond to the antibody specificities. Although it is beyond the scope of assays used in our studies to determine the exact conformational arrangement of each epitope and all amino acids that constitute the epitope, the defining amino acids must be a focal part of the epitope. Public epitopes found exclusively on positive antigens and not on negative antigens are likely not coincidences. For several epitopes defined in our studies, the difference of one aa among alleles of the same antigen, at least one amino acid position can determine whether the allele is positive or negative with the antibody (Figure 5).

We have demonstrated that some antibodies target an epitope on one single antigen (private epitope) or an epitope on a group of two or more antigens (public epitopes). Furthermore, in anti-DQ antisera, immunological responses can produce antibodies to epitopes located on either or both polymorphic chains of the DQ antigens.

The usefulness of epitopes beyond determining correct antibody specificity in sera of transplant patients has been the subject of study recently. Reports on matching donors and recipients or selecting organ donors based on epitope matching are numerous. For example, Duquesnoy reported on HLA epitope-based matching for organ transplantation [35, 36]. Wiebe reported on epitope matching to minimize de novo donor-specific antibodies to improve transplantation outcome [37] and Walton et al. reported on the usefulness of matching at the epitope level which protects against chronic lung allograft dysfunction [38].

## Supplementary Material

Table D. Amino acid positions and number of epitopes defined by each position. Table E Frequency of each amino acid found in all epitope definitions. Figure A Intact HLA class I antigens dissociated to heavy chain, β2m and peptide. Figure B. Epitope 205 shared by the AB-loci antigens A32, A74, B7, B8, B4005, B41, B42, B48, B60, B61, B73, B81 and defined by 109L+131R. Figure C. Epitope 24 shared by the AB-loci Bw4 associated antigens A23, A24, A25, A32, B13,B2705, B37, B38, B44, B47, B49, B51, B52, B53, B57, B58, B59, B63, B77 and defined by either 82L or 83R. Figure D. Epitope 423 shared by the AB-loci Bw4 associated antigens A23, A25, A32, B2705, B37, B38, B44, B47, B49, B51, B52, B53, B57, B58, B59, B63, B77 (A24, B13 Negative) and defined by 83R+144Q+145R. Figure E. Epitope 246 shared by BC-loci antigens B46, B73, Cw1, Cw7, Cw8, Cw9, Cw10, Cw12, Cw14, Cw16 and defined by 76V+80N. Figure F. Epitope 5024 shared by the HLA class I B-locus antigens B7, B42, B54, B55, B56, B67, B81, B82 and defined by 66I+70Q. Reactions strength of the antibody is stronger with the unobstructed epitope after dissociation of the peptide. Figure G. Epitope 5037 shared by the HLA C-locus antigens Cw4, Cw6, Cw17, Cw18 and defined by 73A+77N. Antibody reaction strength increases with the unobstructed epitope after removal of the peptide. Figure H HLA class II DQB epitope 2007 shared by DQ4,5,6 antigens and define by 52P+55R on the beta chain of the DQ antigens. Figure I. Serum from renal transplant patient with mismatch has two antibodies. One antibody targets epitope 2017 (defined by 52H) on the DQA1∗02:01 alpha chain and the other targets epitope 2001 (defined by 52L) on the DQB1∗02:02 beta chain. Figure J. Epitope 2018 shared by the alpha chains of the DQ4, 5, 6 antigens and defined by Glutamine (Q) at position 53. Figure K. Epitope 2002 shared exclusively by the beta chains of the DQ4 antigen and defined by Leucine (L) in position 56. Figure L. Epitope 2010 shared by the beta chains of DQ antigens DQ4, 5, 6, 8, 9 and defined by 45G+46V. Figure M. Epitope 2022 exclusive to DQB1∗05:01 chain on the DQ5 antigen and defined by 125S+126Q. Figure N. Epitope 2006 shared by DQB1∗03:01 (DQ7), DQB1∗03:02 (DQ8), DQB1∗03:03 (DQ9) and defined by Proline (P) at position 55 on the beta chains of the DQ antigens.

## Figures and Tables

**Figure 14:**
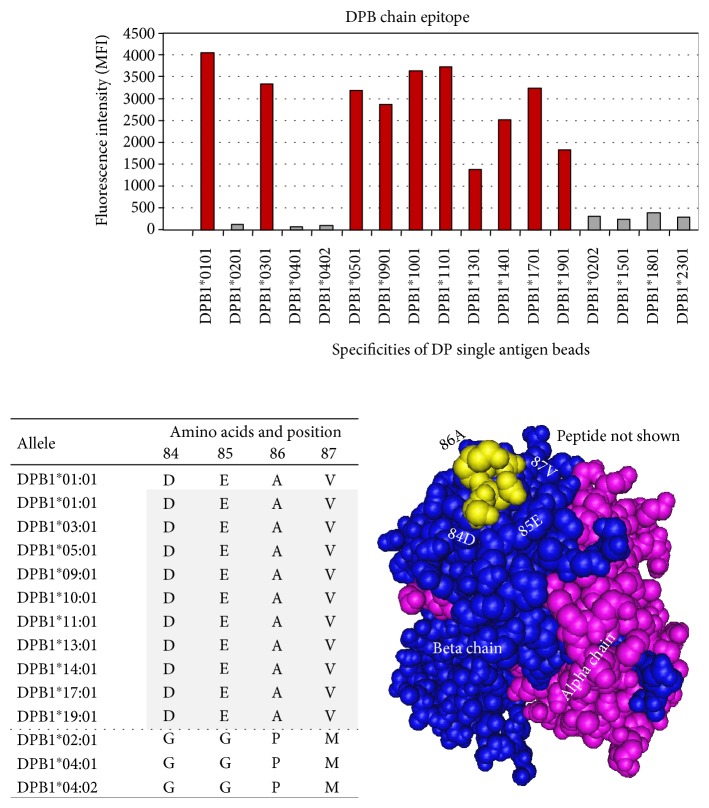
HLA class II DP epitope 4001 shared by DPB chains DPB1^∗^0101, DPB1^∗^0301, DPB1^∗^0501, DPB1^∗^0901, DPB1^∗^1001, DPB1^∗^1101, DPB1^∗^1301, DPB1^∗^1401, DPB1^∗^1701, and DPB1^∗^1901 (red bars) and defined by 84D + 85E + 86A + 87V. Negative antigens that did not share epitope 4001 are shown in (gray bars).

**Table 1:** HLA class I epitope—partial list. Full list of 110 epitopes in supplemental file.

Epitope number	Antigens that share epitope^a^	Amino acid(s) define epitope^b^	A/M	Adsorption rHLA cell line
1	A1, 36	44K/150V/158V/	M	N/A
6	A3	161D	M	N/A
4	A25, 26, 34, 43, 66	(9Y) + 149T/(74D) + 149T^b^	M	N/A
7	B7, 8, 13, 18, 27, 35, 37, 38, 39, 4005, 41, 42, 44, 45, 46, 47, 48, 49, 50, 51, 52, 53, 54, 55, 56, 59, 60, 61, 62, 64, 65, 67, 71, 72, 73, 75, 76, 77, 78, 81, 82	65Q^c^	M	N/A
8	B13	145L/41T + 46A	M	N/A
14	**A1**, 23, 2402, 80, **B76**	166D/167G	A	A2402
16	**A1**, 36, 11, 25, 26, 34, 43, 6601, 80, **B73**	[90D]^c^	A	A8001
17	A2, **B57**, 58	62G	A	B5801
18	A2, 68, 69	142T/145H	A	A6901
**40**	Cw5, 8	177K	A	nn
**222**	A6602, **B7**, 13, 27, 47, 48, 60, 61, 73, 81, **Cw2**, Cw17	163E + 166E/ 163E + 167W	A4	Cw0202
**223**	B7, 13, 27, 47, 48, 60, 61, 81	76E + 163E	A	B0703
**244**	Cw2, 4, 5, 6, 15, 17, 18	77N + 80K	A	Cw1701
**244**	**B35**, 4005, 46, 49, 50, 51, 52, 53, 56, 57, 58, 62, 63, 71, 72, 75, 77, 78, **Cw9**, Cw10	163L + 167W	A	B62 (B1501)/B35
**246**	**B46**, 73, **Cw1**, 7, 8, 9, 10, 12, 14, 16	76V + 80N/73T + 76V + 79R	A/A	Cw1802/nn
**249**	A^∗^2301, A^∗^2402, A^∗^2403, A^∗^2501, A^∗^3201, B^∗^1513, B^∗^1516, B^∗^27052, B^∗^3701, B^∗^3801, B^∗^4402, B^∗^4403, B^∗^4701, B^∗^4901, B^∗^5101, B^∗^5102, B^∗^5201, B^∗^5202, B^∗^5301, B^∗^5701, B^∗^5703, B^∗^5801, B^∗^5901 **B**^∗^1301**&B**^∗^1302***Neg*.**	82L + 145R / 83R + 145R	M	N/A
**250**	A^∗^2301, A^∗^2402, A^∗^2403, A^∗^3201, B^∗^1301, B^∗^1302, B^∗^1513, B^∗^1516, B^∗^27052, B^∗^3701, B^∗^3801, B^∗^4402, B^∗^4403, B^∗^4701, B^∗^4901, B^∗^5101, B^∗^5102, B^∗^5201, B^∗^5202, B^∗^5301, B^∗^5701, B^∗^5703, B^∗^5801, B^∗^5901 ***A2501 Neg.***	82L + 90A/83R + 90A	M	N/A

M designates murine monoclonal antibody, A designates alloantibody, and adsorption rHLA cell line indicates the cell line used to adsorb then elute the antibody; aa: amino acids; nn: not needed; N/A: not applicable; ^a^serological antigens shown, alleles are shown when not all alleles of an antigen are positive (i.e., share epitope); ^b^possible alternative epitopes are separated by “/”; plus sign “+” indicates two or more positions/aa needed to define the epitope; amino acids not exposed at the surface of the HLA molecule are between parentheses; ^c^epitope also shared by C-locus antigens (not shown here) is between square brackets.

**Table 2:** Epitopes on intact antigens targeted by naturally occurring antibodies—partial list. Complete list in supplemental file.

Epitope number	Dissociated antigen(s)	Epitope site	Epitope number	Dissociated antigen(s)	Epitope site
5059	A0101	158V + 163R	5073	B76	163L + 166D
201	A2	43Q + 62G	5075	Cw^∗^0102, 0302, 0303, 0304, 1402, 1802	219W
3	A23, A24	65G	5076	Cw16	193L
31	A30, 31	56R	5077	Cw17	170G
5064	A3002	17S + 76E	5078	Cw7	273S
5066	A6602	149T	5081	Cw9, Cw10	163L + 173K
5068	A80	56E+	5085	B8	(67F) + 131R
406	B2705	65Q + 69A + 80T	5086	Cw6	80K + 90D + (114D)
236	B57, B58	43P + 62G			

Plus sign “+” indicates two or more positions/aa needed to define the epitope; amino acids not exposed at the surface of the HLA molecule are between parentheses.

**Table 3:** Partial list of epitopes on HLA class I antigens. Complete list in supplemental file.

Antigen	Number of epitopes	Epitope number																
A1	11	1	12	13	14	15	16	208	238	241	242	248						
A2	16	2	13	17	18	19	27	32	38	201	210	211	238	242	247	412	422	
A25	19	4	12	16	23	24	27	32	38	209	211	213	214	233	238	241	243	
		247	249	423														
A80	9	13	14	15	16	28	29	208	241	242	A80	9	13	14	15	16	28	29
B13	16	7	8	21	22	24	32	33	43	217	218	222	223	233	235	250	418	
B54	17	7	25	32	33	204	215	216	224	226	228	229	232	233	234	401	402	410
B76	13	7	14	22	25	33	43	211	216	218	227	233	240	403	B76	13	7	14
Cw1	5	32	205	232	246	421												
CW2	5	32	39	205	222	244												
CW4	4	32	205	232	244													
CW9	6	32	39	205	245	246	421											
CW10	6	32	39	205	245	246	421											

**Table 4:** Cryptic (C) epitopes on dissociated class I HLA antigen—partial list. Complete list in supplemental file.

Epitope number	Dissociated antigen(s)	Epitope site	Epitope number	Dissociated antigen(s)	Epitope site^c^
5006	A3002	(152R)	5033	Cw2	(211T)
5007	A31, A33	(73I)	5036	Cw17	(116F) + (143S)
5008	A3401	(63N) + (66K)	5038	Cw6	(9D) + (97W)
5009	A3402	(63N) + (66K) + (156L)	5039	Cw7	(66K) + (99S)
5010	A80	(31S)	5049	A6602	(114Q) + 163E
5027	B8	(9D)	5052	B76	(70N) + 166D
5031	B82	(24S) + (99F)			

^c^Amino acids and their positions on the HLA-dissociated antigens define each epitope. In intact antigens, these amino acids are not exposed at the surface (cryptic).

**Table 5:** MICA epitopes.

Epitope number	MICA antigens sharing epitope	aa/position define epitope^a^	rMICA cells used for adsorption/elution
6001	MICA^∗^001, 012, 018	(24T)	MICA^∗^018
6002	MICA^∗^001, 002, 004, 007, 009, 012, 018, 027	91Q	ND
6003	MICA^∗^004, 009	122V	ND
6004	MICA^∗^027, 004, 009	(36Y)/129V/173E	MICA^∗^004
6005	MICA^∗^017	91R	ND
6006	MICA^∗^004	181R	ND
6007	MICA^∗^027	213I/251R	ND

ND: not done; amino acids not exposed on the surface of the MICA antigen are shown between parentheses; ^a^possible alternative epitope definitions are separated by “/”; epitopes.

**Table 6:** Partial list of HLA class II DR epitopes defined based on aa acid sequence of the beta chain of the antigens. Complete list in supplemental file.

Epitope number	DR antigens sharing epitope	Position/amino acid
1001	DR7, DR9, DR53	4Q
1004	DR4, DR10	11V
1008	DR7	25Q
1017	DR11	58E
1018	DR7, DR8, DR11, DR12, DR13, DR16, DR51, DR103	70D
1028	DR1, DR4, DR7, DR9, DR10, DR11, DR12, DR13, DR14, DR15, DR16, DR51, DR53, DR103	77T
1029	DR7, DR9	78V
1039	DR1, DR7, DR9, DR15, DR16, DR51, DR52, DR53, DR103	140A
1032	DR7, DR8, DR9, DR10, DR11, DR12, DR13, DR14, DR17, DR18, DR52	96H

**Table 7:** Fifteen HLA class II DQ*β* epitopes and three DQ*α* epitopes defined.

Epitope number^a^	DQ antigens sharing epitope	Position/amino acid^b^
2001	DQB2	28S/30S/37I/52L/55L
2002	DQB4	56L
2003	DQB4, DQB5, DQB6, DQB7, DQB8, DQB9	28T/46V/52P
2004	DQB5, DQB6	84E/85V/86A/89G/90I/221Q
2005	DQB7	45E
2006	DQB7, DQB8, DQB9	55P
2007	DQB4, DQB5, DQB6	52P + 55R
2008	DQB2, DQB5, DQB7, DQB8, DQB9	(9Y + 11F)
2009	DQB2, DQB4, DQB5, DQB6, DQB8, DQB9	34R + 45G
2010	DQB4, DQB5, DQB6, DQB8, DQB9	45G + 46V
2011	DQB5, DQB0601	38V + 46V
2012	DQB8, DQB9	45G + 55P
2013	DQB2, DQB4, DQB7, DQB8, DQB9	84Q/85L/86E/87L/89T/220H/221H
2014	DQB4, DQB7, DQB8, DQB9	77T + 84Q/77T + 85L/77T + 86E/77T + 87L/182N
2015	DQB5	70G + 71A/116I/125S
2017	DQA1∗0201	47K/52H/54L
2018	DQA1∗04/DQA1∗05/DQA1∗06	40G/47C
2019	DQA1∗03	26S/47Q/56R/187T

^a^Epitope 2008 defined using mAb; ^b^possible alternative epitopes are separated by “/”; epitopes that are defined by more than a single position/aa are separated by “+”; amino acids not exposed at the surface of the HLA molecule are between parentheses.

**Table 8:** Number and epitopes on HLA class II DQA1 and DQB1 antigens.

Antigen	Number of epitopes	Epitopes							
DQA1^∗^0201	1	2017							
DQA1^∗^03	1	2019							
DQA1^∗^04	1	2018							
DQ2	4	2001	2008	2009	2013				
DQ4	7	2002	2003	2007	2009	2010	2013	2014	
DQ5	8	2003	2004	2007	2008	2009	2010	2011	2015
DQ0601	6	2003	2004	2007	2009	2010	2011		
DQ7	6	2003	2005	2006	2008	2013	2014		
DQ8	8	2003	2006	2008	2009	2010	2012	2013	2014
DQ9	8	2003	2006	2008	2009	2010	2012	2013	2014

**Table 9:** HLA class II DP epitopes.

Epitope number	DP antigens sharing epitope	Position/amino acid^a^
4001	DPB1^∗^0101, DPB1^∗^0301, DPB1^∗^0501, DPB1^∗^0901, DPB1^∗^1001, DPB1^∗^1101, DPB1^∗^1301, DPB1^∗^1401, DPB1^∗^1701, DPB1^∗^1901	84D + 85E + 86A + −87V
4002	DPB1^∗^0301, DPB1^∗^0901, DPB1^∗^1401, DPB1^∗^1701	55D + 56E + -57D
4003	DPB1^∗^0201, DPB1^∗^0402, DPB1^∗^1001, DPB1^∗^1801	55D + 56E + -57E
4004	DPB1^∗^1101, DPB1^∗^1501	(33Q)
4005	DPB1^∗^0201, DPB1^∗^0401, DPB1^∗^0402	84G + 85G + 86P + 87M

^a^Epitopes defined by more than a single position/aa are separated by “+”; amino acids not exposed at the surface of the HLA molecule are between parentheses.
