# Supplementary material for: HLA Epitopes: The Targets of Monoclonal and Alloantibodies Defined
Source: J Immunol Res. 2017 May 24;2017:3406230. doi: 10.1155/2017/3406230 (PMC5463109; doi:10.1155/2017/3406230)
Supplement: Supplementary file 1 — Table D. Amino acid positions and number of epitopes defined by each position. Table E Frequency of each amino acid found in all epitope definitions. Figure A Intact HLA class I antigens dissociated to heavy chain, β2m and peptide. Figure B. Epitope 205 shared by the AB-loci antigens A32, A74, B7, B8, B4005, B41, B42, B48, B60, B61, B73, B81 and defined by 109L+131R. Figure C. Epitope 24 shared by the AB-loci Bw4 associated antigens A23, A24, A25, A32, B13,B2705, B37, B38, B44, B47, B49, B51, B52, B53, B57, B58, B59, B63, B77 and defined by either 82L or 83R. Figure D. Epitope 423 shared by the AB-loci Bw4 associated antigens A23, A25, A32, B2705, B37, B38, B44, B47, B49, B51, B52, B53, B57, B58, B59, B63, B77 (A24, B13 Negative) and defined by 83R+144Q+145R. Figure E. Epitope 246 shared by BC-loci antigens B46, B73, Cw1, Cw7, Cw8, Cw9, Cw10, Cw12, Cw14, Cw16 and defined by 76V+80N. Figure F. Epitope 5024 shared by the HLA class I B-locus antigens B7, B42, B54, B55, B56, B67, B81, B82 and defined by 66I+70Q. Reactions strength of the antibody is stronger with the unobstructed epitope after dissociation of the peptide. Figure G. Epitope 5037 shared by the HLA C-locus antigens Cw4, Cw6, Cw17, Cw18 and defined by 73A+77N. Antibody reaction strength increases with the unobstructed epitope after removal of the peptide. Figure H HLA class II DQB epitope 2007 shared by DQ4,5,6 antigens and define by 52P+55R on the beta chain of the DQ antigens. Figure I. Serum from renal transplant patient with mismatch has two antibodies. One antibody targets epitope 2017 (defined by 52H) on the DQA1∗02:01 alpha chain and the other targets epitope 2001 (defined by 52L) on the DQB1∗02:02 beta chain. Figure J. Epitope 2018 shared by the alpha chains of the DQ4, 5, 6 antigens and defined by Glutamine (Q) at position 53. Figure K. Epitope 2002 shared exclusively by the beta chains of the DQ4 antigen and defined by Leucine (L) in position 56. Figure L. Epitope 2010 shared by the beta chains of [file 3406230.f1.pptx]

## Slide 1
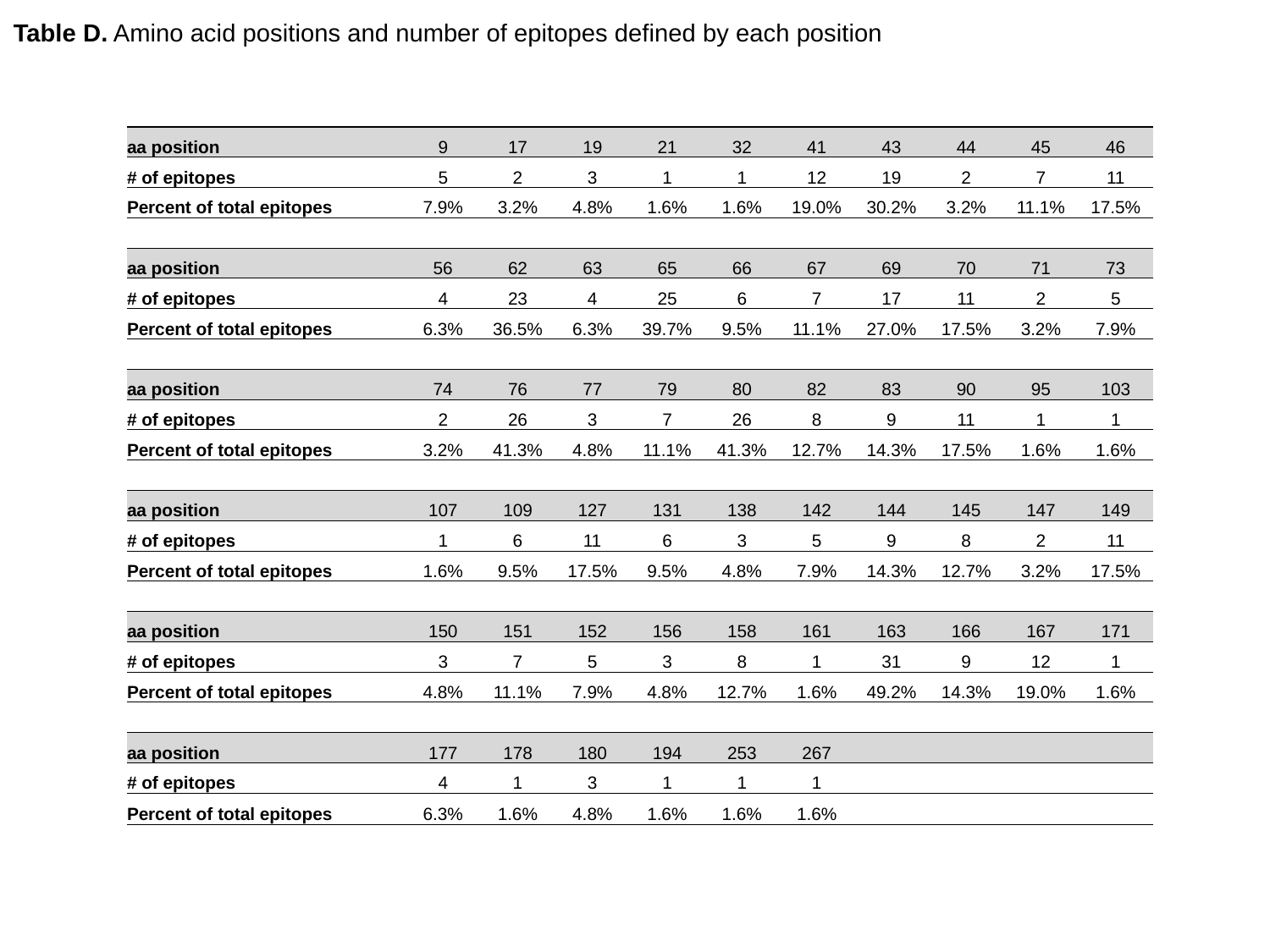

Table D. Amino acid positions and number of epitopes defined by each position
| aa position | 9 | 17 | 19 | 21 | 32 | 41 | 43 | 44 | 45 | 46 |
| --- | --- | --- | --- | --- | --- | --- | --- | --- | --- | --- |
| # of epitopes | 5 | 2 | 3 | 1 | 1 | 12 | 19 | 2 | 7 | 11 |
| Percent of total epitopes | 7.9% | 3.2% | 4.8% | 1.6% | 1.6% | 19.0% | 30.2% | 3.2% | 11.1% | 17.5% |
| | | | | | | | | | | |
| aa position | 56 | 62 | 63 | 65 | 66 | 67 | 69 | 70 | 71 | 73 |
| # of epitopes | 4 | 23 | 4 | 25 | 6 | 7 | 17 | 11 | 2 | 5 |
| Percent of total epitopes | 6.3% | 36.5% | 6.3% | 39.7% | 9.5% | 11.1% | 27.0% | 17.5% | 3.2% | 7.9% |
| | | | | | | | | | | |
| aa position | 74 | 76 | 77 | 79 | 80 | 82 | 83 | 90 | 95 | 103 |
| # of epitopes | 2 | 26 | 3 | 7 | 26 | 8 | 9 | 11 | 1 | 1 |
| Percent of total epitopes | 3.2% | 41.3% | 4.8% | 11.1% | 41.3% | 12.7% | 14.3% | 17.5% | 1.6% | 1.6% |
| | | | | | | | | | | |
| aa position | 107 | 109 | 127 | 131 | 138 | 142 | 144 | 145 | 147 | 149 |
| # of epitopes | 1 | 6 | 11 | 6 | 3 | 5 | 9 | 8 | 2 | 11 |
| Percent of total epitopes | 1.6% | 9.5% | 17.5% | 9.5% | 4.8% | 7.9% | 14.3% | 12.7% | 3.2% | 17.5% |
| | | | | | | | | | | |
| aa position | 150 | 151 | 152 | 156 | 158 | 161 | 163 | 166 | 167 | 171 |
| # of epitopes | 3 | 7 | 5 | 3 | 8 | 1 | 31 | 9 | 12 | 1 |
| Percent of total epitopes | 4.8% | 11.1% | 7.9% | 4.8% | 12.7% | 1.6% | 49.2% | 14.3% | 19.0% | 1.6% |
| | | | | | | | | | | |
| aa position | 177 | 178 | 180 | 194 | 253 | 267 | | | | |
| # of epitopes | 4 | 1 | 3 | 1 | 1 | 1 | | | | |
| Percent of total epitopes | 6.3% | 1.6% | 4.8% | 1.6% | 1.6% | 1.6% | | | | |
