# Supplementary material for: HLA Epitopes: The Targets of Monoclonal and Alloantibodies Defined
Source: J Immunol Res. 2017 May 24;2017:3406230. doi: 10.1155/2017/3406230 (PMC5463109; doi:10.1155/2017/3406230)
Supplement: Supplementary file 2 [file 3406230.f2.pptx]

## Slide 1
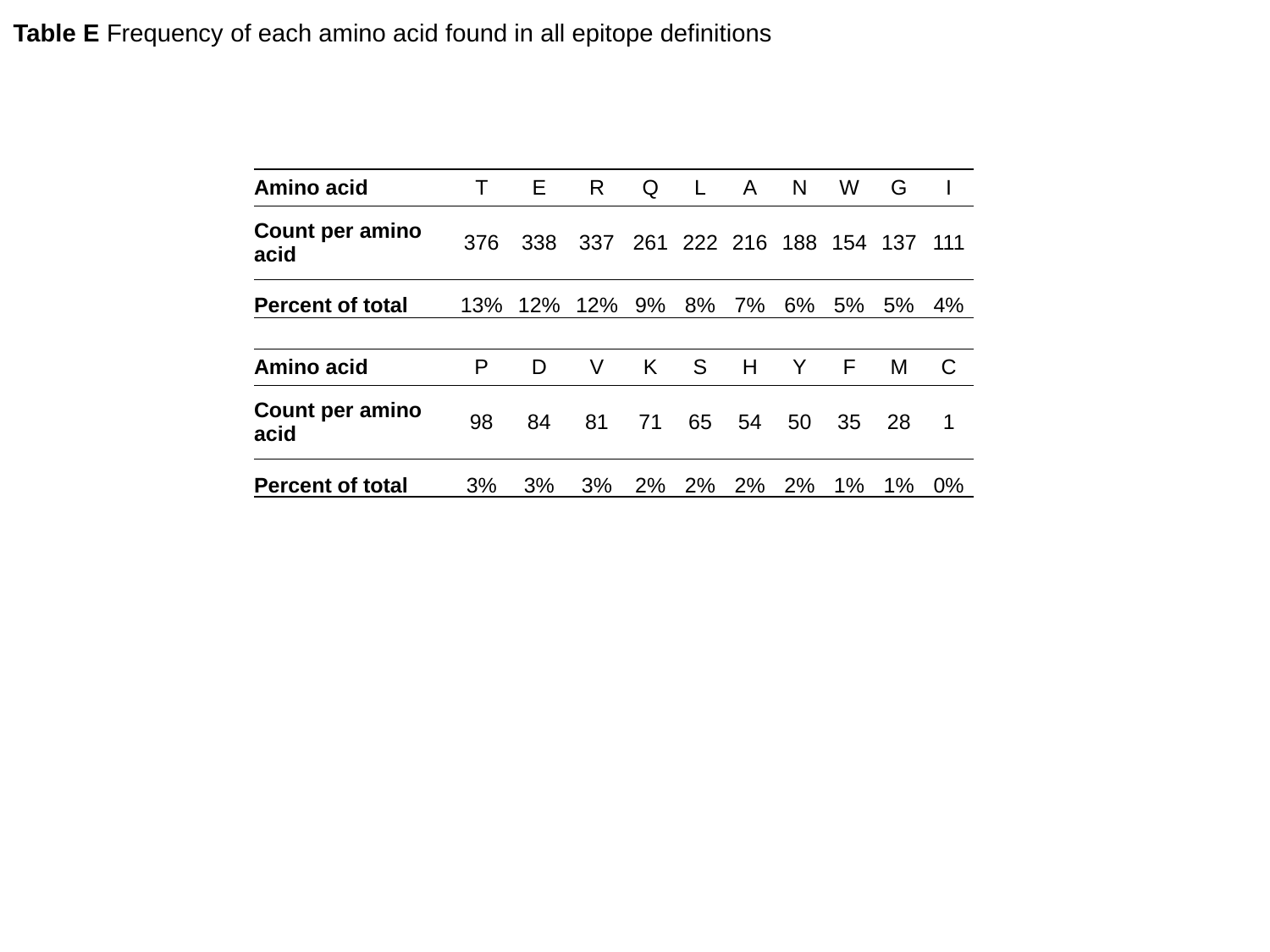

Table E Frequency of each amino acid found in all epitope definitions
| Amino acid | T | E | R | Q | L | A | N | W | G | I |
| --- | --- | --- | --- | --- | --- | --- | --- | --- | --- | --- |
| Count per amino acid | 376 | 338 | 337 | 261 | 222 | 216 | 188 | 154 | 137 | 111 |
| Percent of total | 13% | 12% | 12% | 9% | 8% | 7% | 6% | 5% | 5% | 4% |
| | | | | | | | | | | |
| Amino acid | P | D | V | K | S | H | Y | F | M | C |
| Count per amino acid | 98 | 84 | 81 | 71 | 65 | 54 | 50 | 35 | 28 | 1 |
| Percent of total | 3% | 3% | 3% | 2% | 2% | 2% | 2% | 1% | 1% | 0% |
