# Supplementary material for: HLA Epitopes: The Targets of Monoclonal and Alloantibodies Defined
Source: J Immunol Res. 2017 May 24;2017:3406230. doi: 10.1155/2017/3406230 (PMC5463109; doi:10.1155/2017/3406230)
Supplement: Supplementary file 3 [file 3406230.f3.pptx]

## Slide 1
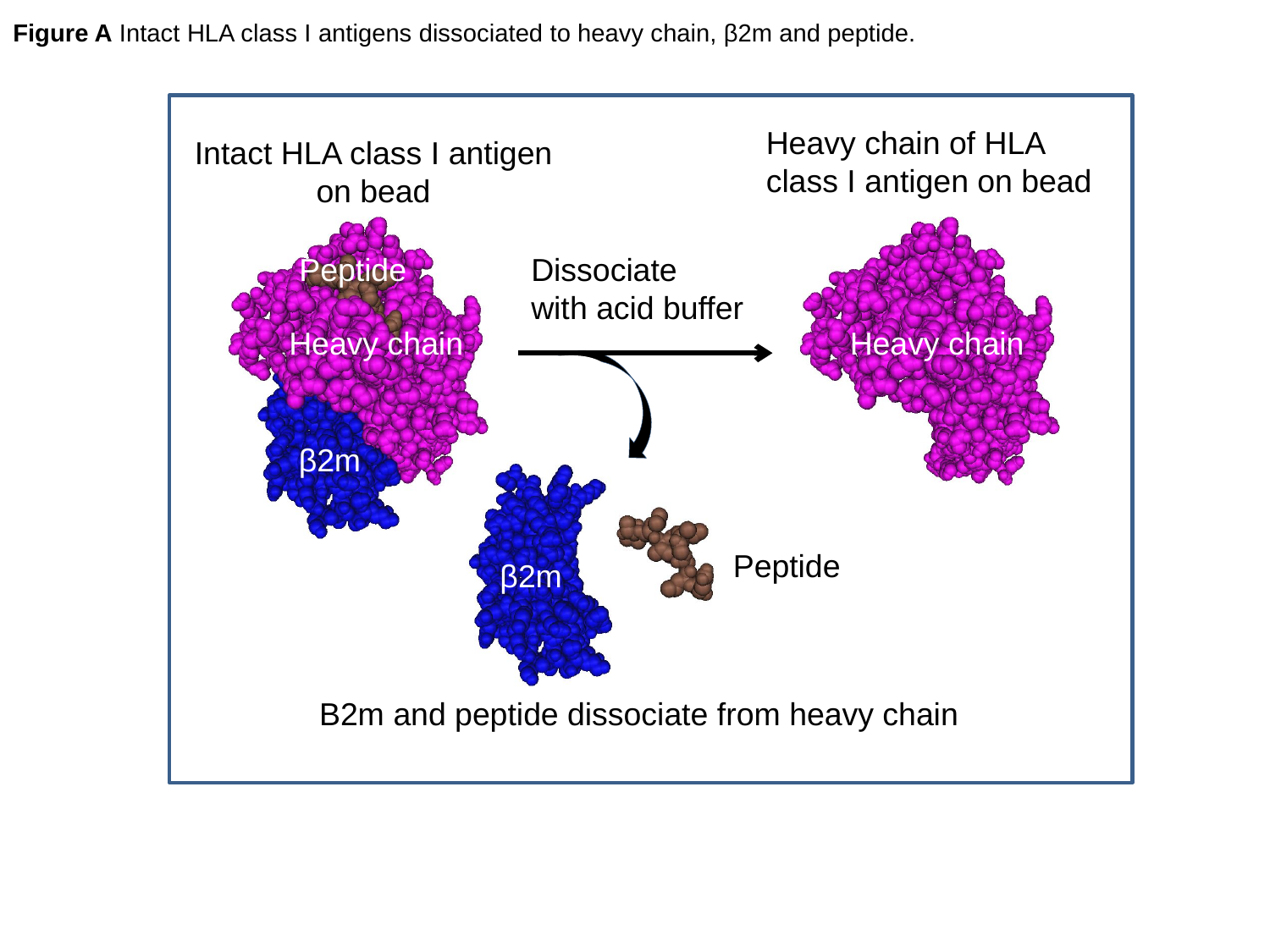

Figure A Intact HLA class I antigens dissociated to heavy chain, β2m and peptide.
Heavy chain of HLA
class I antigen on bead
Intact HLA class I antigen
on bead
Peptide
Dissociate
with acid buffer
Heavy chain
Heavy chain
β2m
Peptide
β2m
Β2m and peptide dissociate from heavy chain
