# Supplementary material for: HLA Epitopes: The Targets of Monoclonal and Alloantibodies Defined
Source: J Immunol Res. 2017 May 24;2017:3406230. doi: 10.1155/2017/3406230 (PMC5463109; doi:10.1155/2017/3406230)
Supplement: Supplementary file 4 [file 3406230.f4.pptx]

## Slide 1
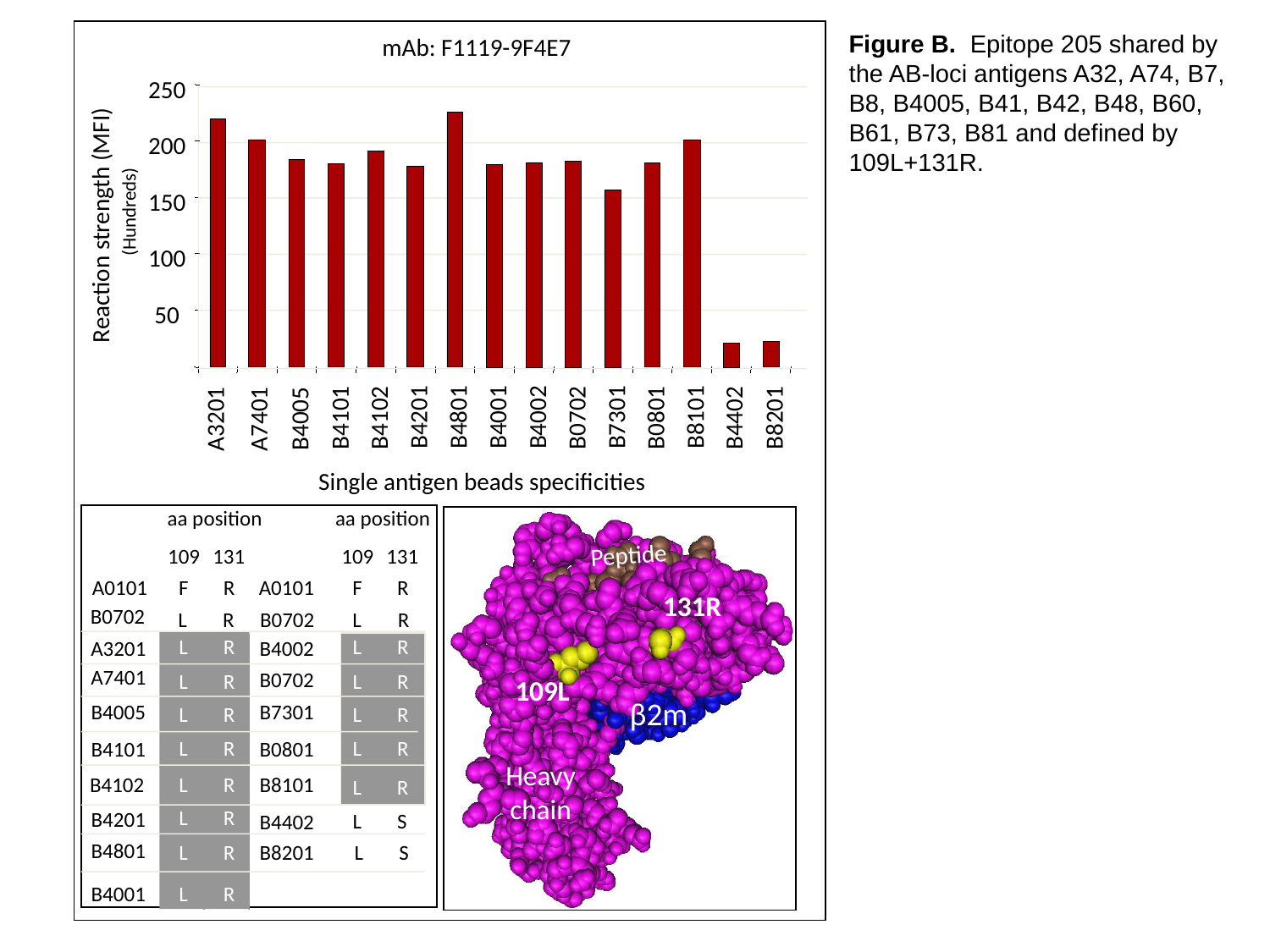

mAb: F1119-9F4E7
250
200
150
(Hundreds)
Reaction strength (MFI)
100
50
B4201
B4801
B4001
B7301
B8101
B4002
B4101
B0702
B4102
B0801
B4402
B8201
A3201
A7401
B4005
Single antigen beads specificities
aa position
aa position
Peptide
109
131
109
131
A0101
F
R
A0101
F
R
131R
L
R
L
R
B0702
L
R
B0702
L
R
L
R
L
R
A3201
B4002
A7401
109L
B0702
L
R
L
R
β2m
B4005
B7301
L
R
L
R
L
R
L
R
B4101
B0801
Heavy
chain
L
R
B8101
B4102
L
R
L
R
B4201
L
S
B4402
B4801
L
R
B8201
L
S
B4001
L
R
Figure B. Epitope 205 shared by the AB-loci antigens A32, A74, B7, B8, B4005, B41, B42, B48, B60, B61, B73, B81 and defined by 109L+131R.
