# Supplementary material for: HLA Epitopes: The Targets of Monoclonal and Alloantibodies Defined
Source: J Immunol Res. 2017 May 24;2017:3406230. doi: 10.1155/2017/3406230 (PMC5463109; doi:10.1155/2017/3406230)
Supplement: Supplementary file 5 [file 3406230.f5.pptx]

## Slide 1
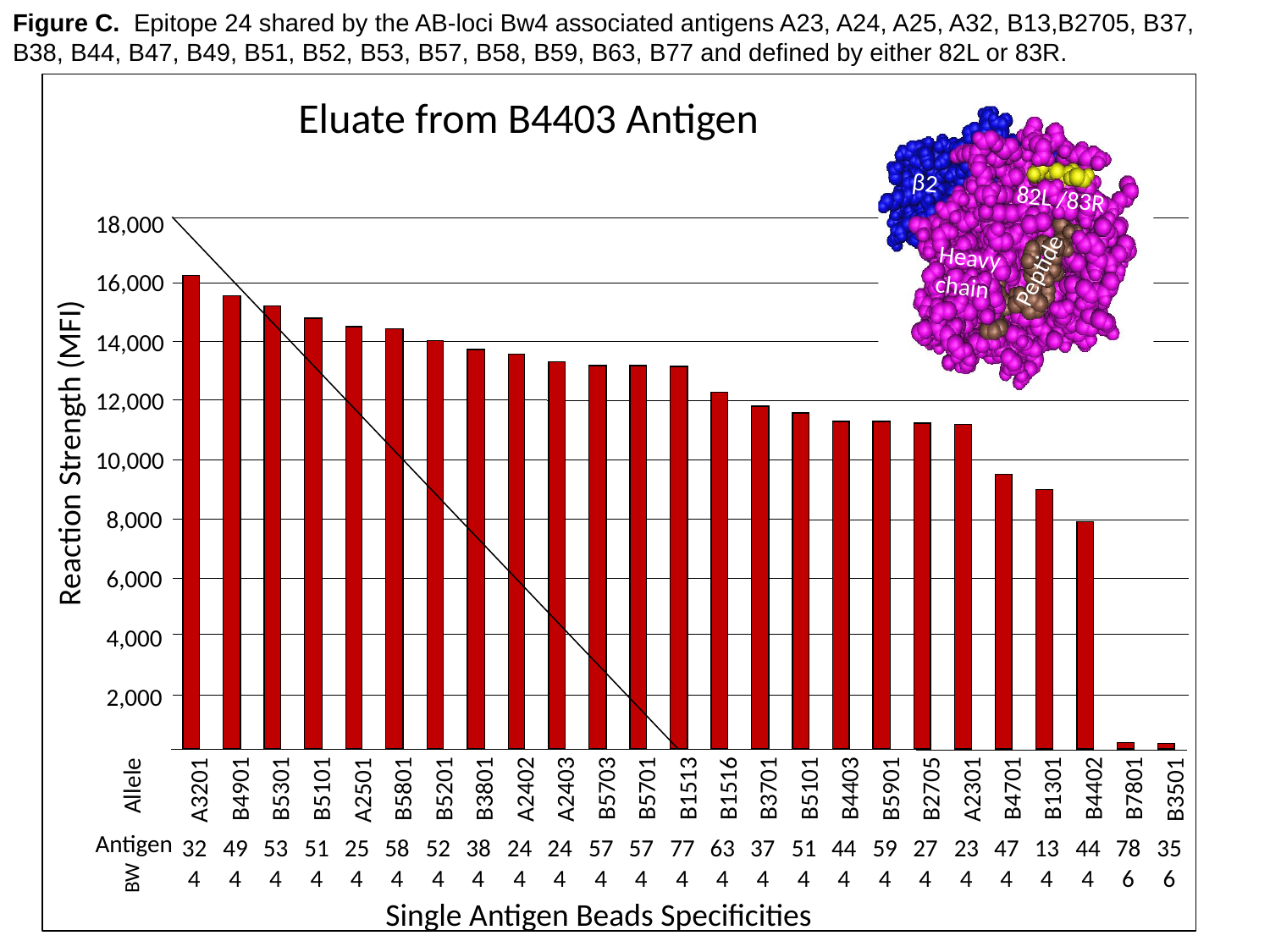

Figure C. Epitope 24 shared by the AB-loci Bw4 associated antigens A23, A24, A25, A32, B13,B2705, B37, B38, B44, B47, B49, B51, B52, B53, B57, B58, B59, B63, B77 and defined by either 82L or 83R.
Eluate from B4403 Antigen
β2
82L /83R
Heavy
chain
Peptide
18,000
16,000
14,000
12,000
Reaction Strength (MFI)
10,000
8,000
6,000
4,000
2,000
Allele
B4701
B1301
B4402
B7801
B5703
B5701
B1513
B1516
B3701
B5101
B4403
B4901
B5301
B5101
B5801
B5201
B3801
B5901
B2705
A2402
A2403
A2301
B3501
A3201
A2501
Antigen
32
49
53
51
25
58
52
38
24
24
57
57
77
63
37
51
44
59
27
23
47
13
44
78
35
4
4
4
4
4
4
4
4
4
4
4
4
4
4
4
4
4
4
4
4
4
4
4
6
6
BW
Single Antigen Beads Specificities
