# Supplementary material for: HLA Epitopes: The Targets of Monoclonal and Alloantibodies Defined
Source: J Immunol Res. 2017 May 24;2017:3406230. doi: 10.1155/2017/3406230 (PMC5463109; doi:10.1155/2017/3406230)
Supplement: Supplementary file 6 [file 3406230.f6.pptx]

## Slide 1
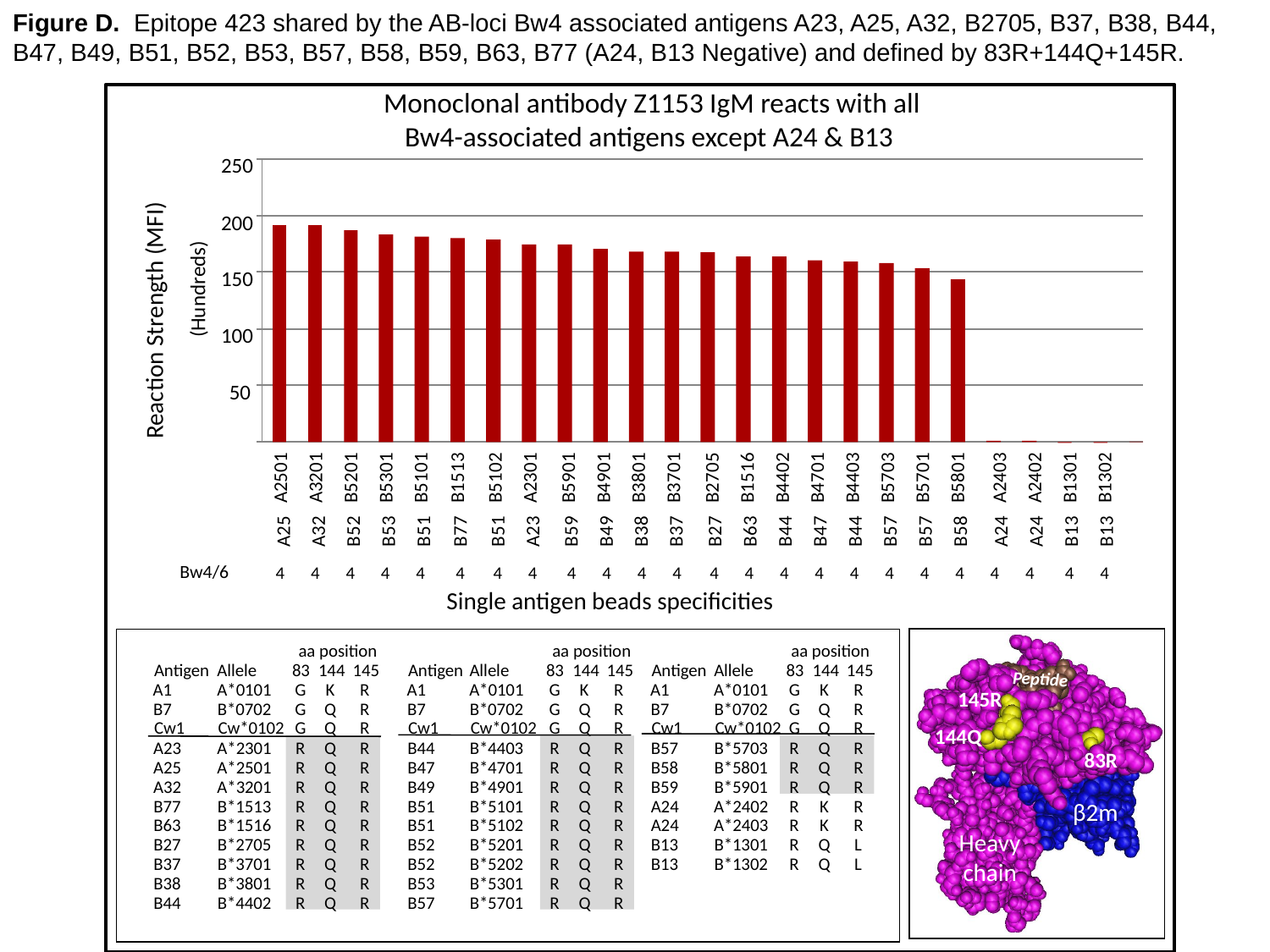

Figure D. Epitope 423 shared by the AB-loci Bw4 associated antigens A23, A25, A32, B2705, B37, B38, B44, B47, B49, B51, B52, B53, B57, B58, B59, B63, B77 (A24, B13 Negative) and defined by 83R+144Q+145R.
Monoclonal antibody Z1153 IgM reacts with all
Bw4-associated antigens except A24 & B13
250
200
150
(Hundreds)
Reaction Strength (MFI)
100
50
A2501
A3201
B5201
B5301
B5101
B1513
B5102
A2301
B5901
B4901
B3801
B3701
B2705
B1516
B4402
B4701
B4403
B5703
B5701
B5801
A2403
A2402
B1301
B1302
A25
A32
B52
B53
B51
B77
B51
A23
B59
B49
B38
B37
B27
B63
B44
B47
B44
B57
B57
B58
A24
A24
B13
B13
Bw4/6
4
4
4
4
4
4
4
4
4
4
4
4
4
4
4
4
4
4
4
4
4
4
4
4
Single antigen beads specificities
aa position
aa position
aa position
Antigen
Allele
83
144
145
Antigen
Allele
83
144
145
Antigen
Allele
83
144
145
Peptide
A1
A*0101
G
K
R
A1
A*0101
G
K
R
A1
A*0101
G
K
R
145R
B7
B*0702
G
Q
R
B7
B*0702
G
Q
R
B7
B*0702
G
Q
R
Cw1
Cw*0102
G
Q
R
Cw1
Cw*0102
G
Q
R
Cw1
Cw*0102
G
Q
R
144Q
A23
A*2301
R
Q
R
B44
B*4403
R
Q
R
B57
B*5703
R
Q
R
83R
A25
A*2501
R
Q
R
B47
B*4701
R
Q
R
B58
B*5801
R
Q
R
A32
A*3201
R
Q
R
B49
B*4901
R
Q
R
B59
B*5901
R
Q
R
β2m
B77
B*1513
R
Q
R
B51
B*5101
R
Q
R
A24
A*2402
R
K
R
B63
B*1516
R
Q
R
B51
B*5102
R
Q
R
A24
A*2403
R
K
R
Heavy
chain
B27
B*2705
R
Q
R
B52
B*5201
R
Q
R
B13
B*1301
R
Q
L
B37
B*3701
R
Q
R
B52
B*5202
R
Q
R
B13
B*1302
R
Q
L
B38
B*3801
R
Q
R
B53
B*5301
R
Q
R
B44
B*4402
R
Q
R
B57
B*5701
R
Q
R
