# Supplementary material for: HLA Epitopes: The Targets of Monoclonal and Alloantibodies Defined
Source: J Immunol Res. 2017 May 24;2017:3406230. doi: 10.1155/2017/3406230 (PMC5463109; doi:10.1155/2017/3406230)
Supplement: Supplementary file 7 [file 3406230.f7.pptx]

## Slide 1
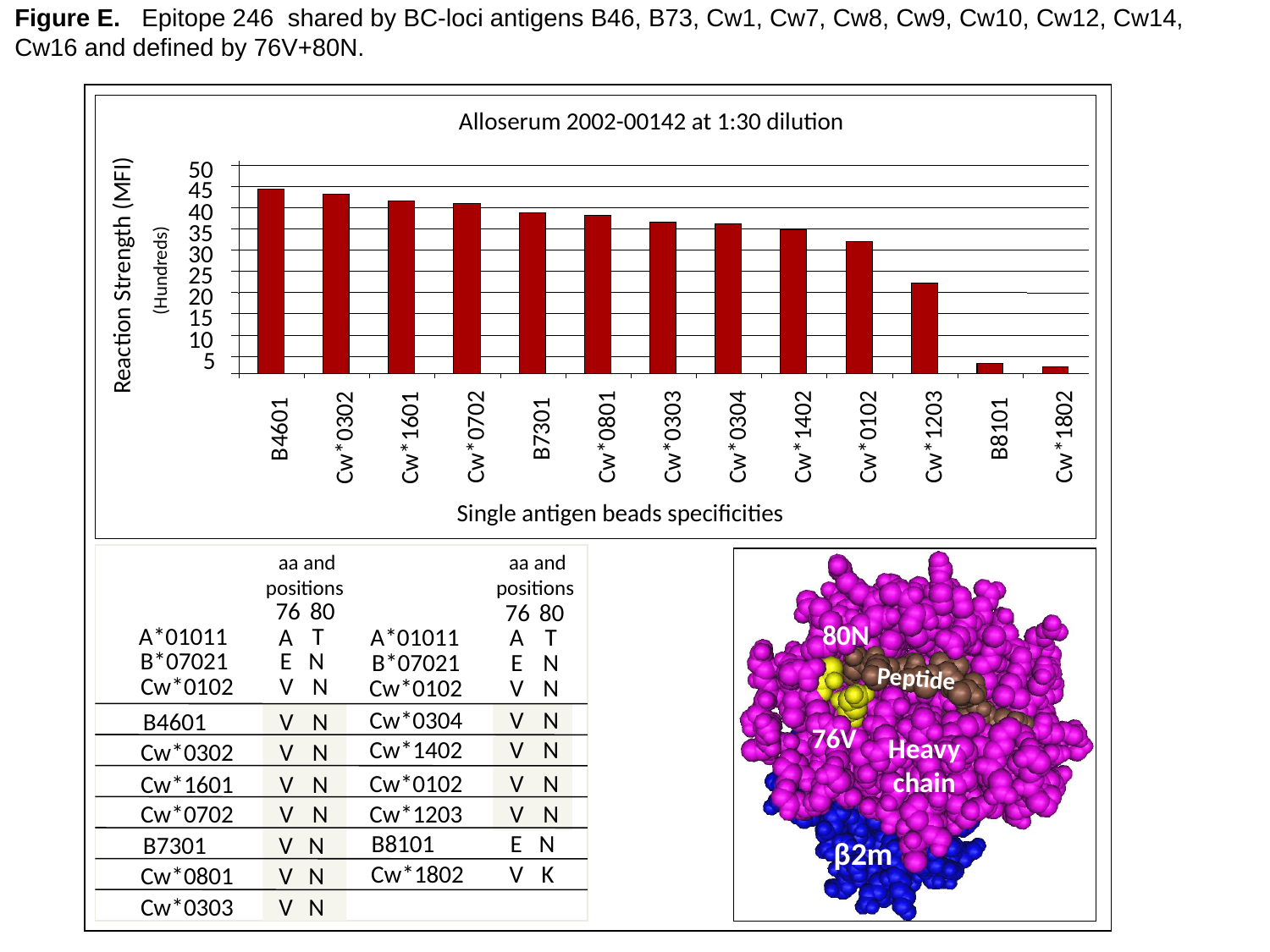

Figure E. Epitope 246 shared by BC-loci antigens B46, B73, Cw1, Cw7, Cw8, Cw9, Cw10, Cw12, Cw14,
Cw16 and defined by 76V+80N.
Alloserum 2002-00142 at 1:30 dilution
50
45
40
35
30
(Hundreds)
25
Reaction Strength (MFI)
20
15
10
5
B8101
B7301
B4601
Cw*1802
Cw*0304
Cw*1402
Cw*0702
Cw*0801
Cw*0303
Cw*0102
Cw*1203
Cw*0302
Cw*1601
Single antigen beads specificities
aa and
positions
aa and
positions
76
80
76
80
80N
A*01011
T
A
A*01011
A
T
B*07021
E
N
B*07021
E
N
Peptide
Cw*0102
V
N
Cw*0102
V
N
Cw*0304
V
N
B4601
V
N
76V
Heavy
chain
Cw*1402
V
N
Cw*0302
V
N
Cw*0102
V
N
Cw*1601
V
N
Cw*0702
V
N
Cw*1203
V
N
B8101
E
N
β2m
B7301
V
N
Cw*1802
V
K
Cw*0801
V
N
Cw*0303
V
N
