# Supplementary material for: HLA Epitopes: The Targets of Monoclonal and Alloantibodies Defined
Source: J Immunol Res. 2017 May 24;2017:3406230. doi: 10.1155/2017/3406230 (PMC5463109; doi:10.1155/2017/3406230)
Supplement: Supplementary file 8 [file 3406230.f8.pptx]

## Slide 1
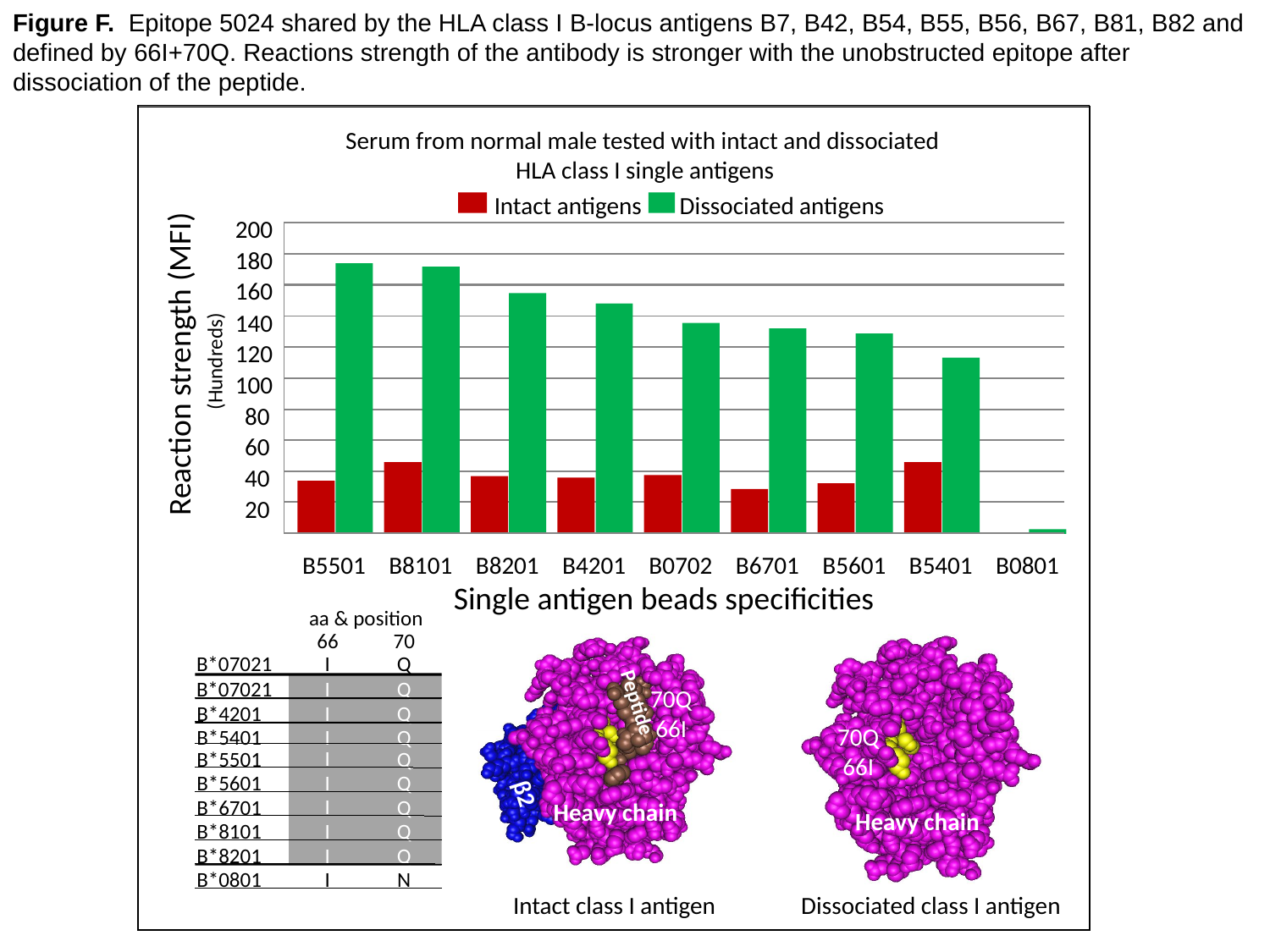

Figure F. Epitope 5024 shared by the HLA class I B-locus antigens B7, B42, B54, B55, B56, B67, B81, B82 and defined by 66I+70Q. Reactions strength of the antibody is stronger with the unobstructed epitope after dissociation of the peptide.
Serum from normal male tested with intact and dissociated
HLA class I single antigens
Intact antigens
Dissociated antigens
200
180
160
140
120
Reaction strength (MFI)
(Hundreds)
100
80
60
40
20
B5501
B8101
B8201
B4201
B0702
B6701
B5601
B5401
B0801
Single antigen beads specificities
aa & position
66
70
B*07021
I
Q
B*07021
I
Q
Peptide
I
B*4201
Q
70Q
66I
B*5401
I
Q
I
B*5501
Q
B*5601
I
Q
β2
Heavy chain
I
B*6701
Q
Heavy chain
B*8101
I
Q
B*8201
I
Q
I
B*0801
N
Intact class I antigen
Dissociated class I antigen
70Q
66I
