# Supplementary material for: HLA Epitopes: The Targets of Monoclonal and Alloantibodies Defined
Source: J Immunol Res. 2017 May 24;2017:3406230. doi: 10.1155/2017/3406230 (PMC5463109; doi:10.1155/2017/3406230)
Supplement: Supplementary file 9 [file 3406230.f9.pptx]

## Slide 1
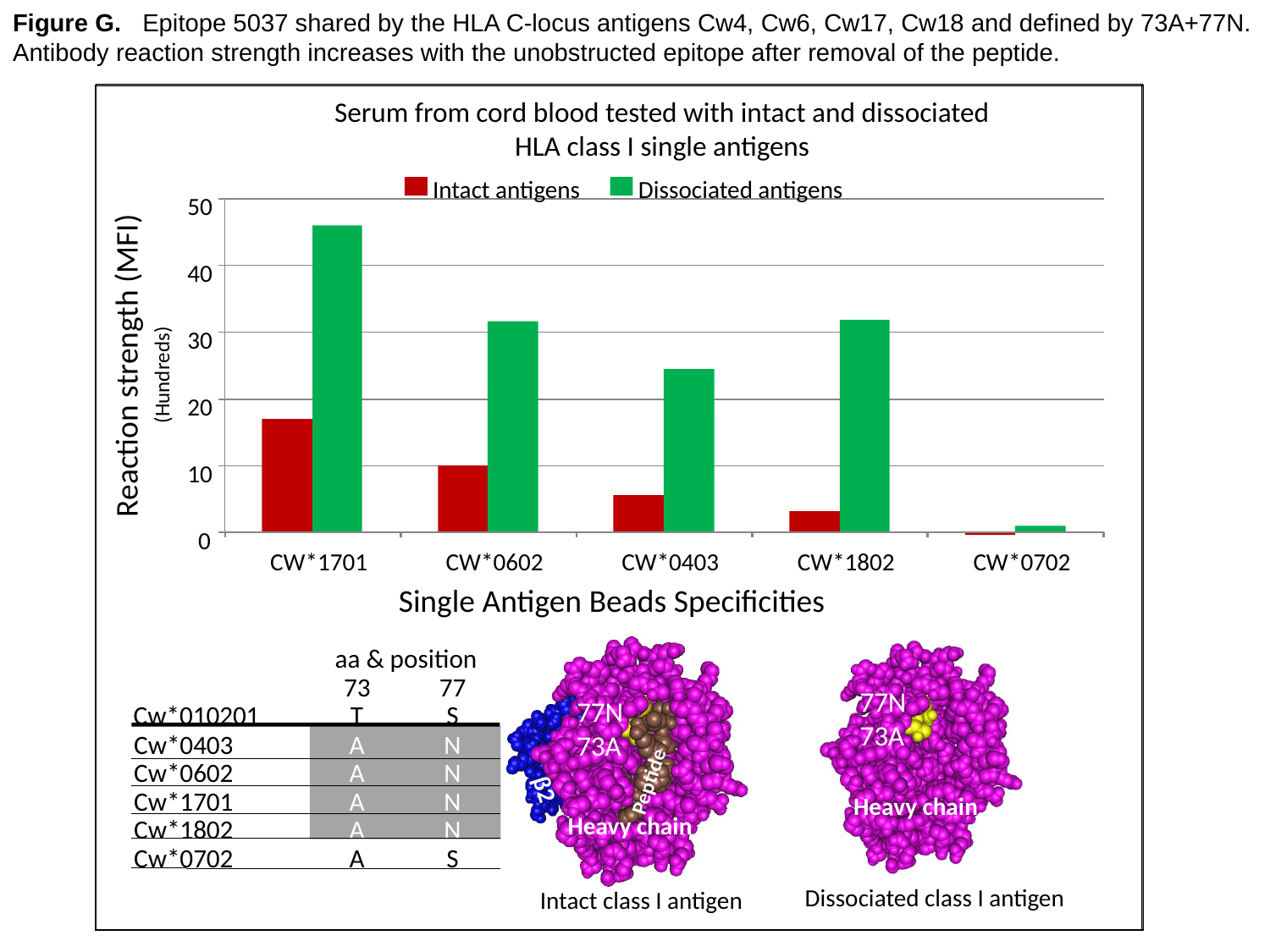

Figure G. Epitope 5037 shared by the HLA C-locus antigens Cw4, Cw6, Cw17, Cw18 and defined by 73A+77N. Antibody reaction strength increases with the unobstructed epitope after removal of the peptide.
Serum from cord blood tested with intact and dissociated
HLA class I single antigens
Intact antigens
Dissociated antigens
50
40
30
Reaction strength (MFI)
(Hundreds)
20
10
0
CW*1701
CW*0602
CW*0403
CW*1802
CW*0702
Single Antigen Beads Specificities
aa & position
73
77
77N
73A
Cw*010201
T
S
Cw*0403
A
N
Cw*0602
A
N
Peptide
β2
Heavy chain
Cw*1701
A
N
Heavy chain
Cw*1802
A
N
Cw*0702
A
S
Dissociated class I antigen
Intact class I antigen
77N
73A
