# Supplementary material for: HLA Epitopes: The Targets of Monoclonal and Alloantibodies Defined
Source: J Immunol Res. 2017 May 24;2017:3406230. doi: 10.1155/2017/3406230 (PMC5463109; doi:10.1155/2017/3406230)
Supplement: Supplementary file 10 [file 3406230.f10.pptx]

## Slide 1
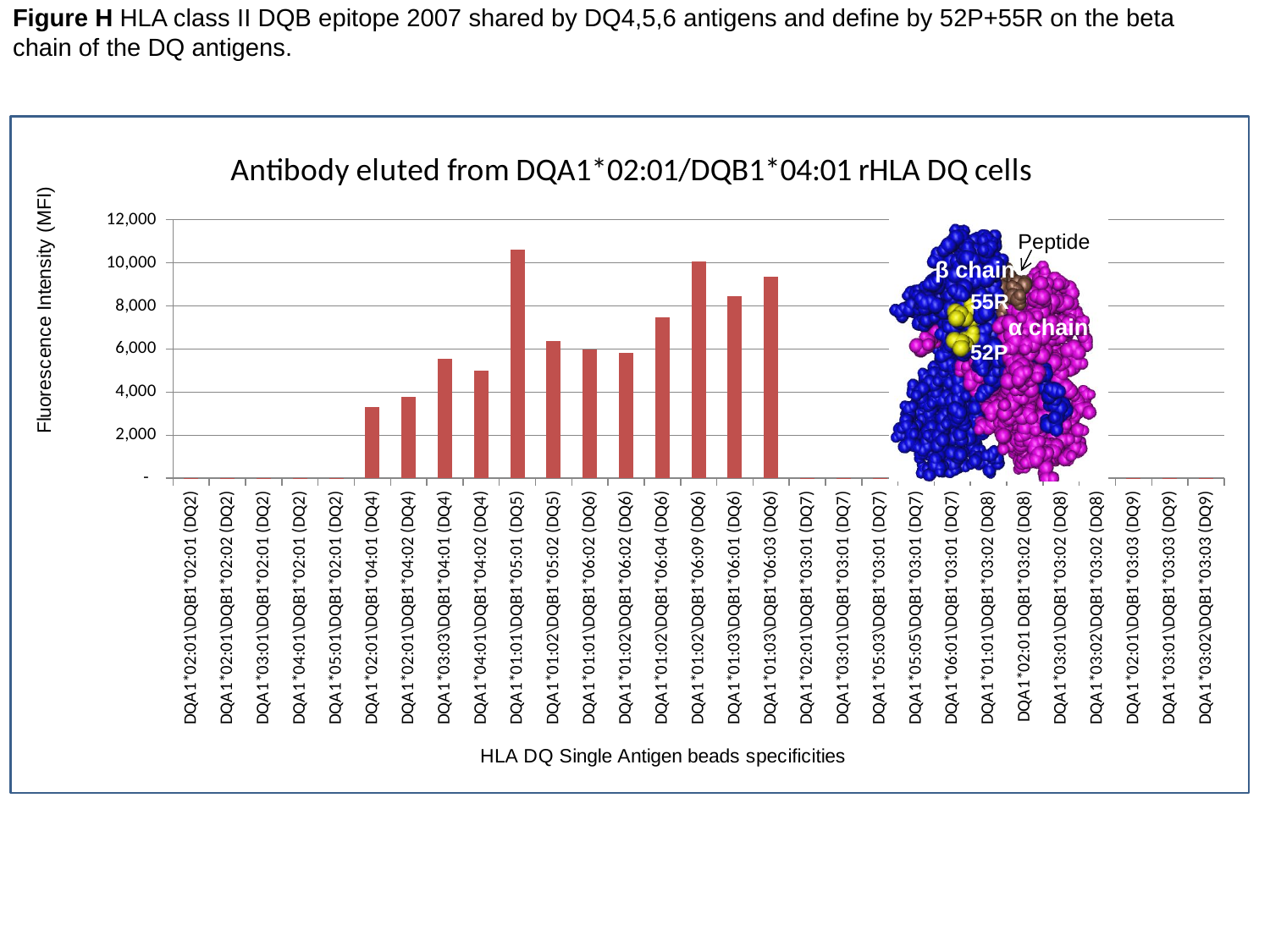

Figure H HLA class II DQB epitope 2007 shared by DQ4,5,6 antigens and define by 52P+55R on the beta chain of the DQ antigens.
### Chart: Antibody eluted from DQA1*02:01/DQB1*04:01 rHLA DQ cells
| Category | #23 RC2265 A DQ4-DQ401.201 |
|---|---|
| DQA1*02:01\DQB1*02:01 (DQ2) | 8.6 |
| DQA1*02:01\DQB1*02:02 (DQ2) | 14.0661157024793 |
| DQA1*03:01\DQB1*02:01 (DQ2) | 14.055555555555614 |
| DQA1*04:01\DQB1*02:01 (DQ2) | 12.9459459459459 |
| DQA1*05:01\DQB1*02:01 (DQ2) | 10.3189655172414 |
| DQA1*02:01\DQB1*04:01 (DQ4) | 3291.67889908257 |
| DQA1*02:01\DQB1*04:02 (DQ4) | 3769.10743801653 |
| DQA1*03:03\DQB1*04:01 (DQ4) | 5561.485507246376 |
| DQA1*04:01\DQB1*04:02 (DQ4) | 4988.924812030076 |
| DQA1*01:01\DQB1*05:01 (DQ5) | 10623.608695652192 |
| DQA1*01:02\DQB1*05:02 (DQ5) | 6373.63636363636 |
| DQA1*01:01\DQB1*06:02 (DQ6) | 5960.9219858156 |
| DQA1*01:02\DQB1*06:02 (DQ6) | 5835.620967741944 |
| DQA1*01:02\DQB1*06:04 (DQ6) | 7489.610169491539 |
| DQA1*01:02\DQB1*06:09 (DQ6) | 10061.053435114507 |
| DQA1*01:03\DQB1*06:01 (DQ6) | 8449.47169811321 |
| DQA1*01:03\DQB1*06:03 (DQ6) | 9359.640625 |
| DQA1*02:01\DQB1*03:01 (DQ7) | 13.401574803149607 |
| DQA1*03:01\DQB1*03:01 (DQ7) | 23.649122807017488 |
| DQA1*05:03\DQB1*03:01 (DQ7) | 13.4700854700855 |
| DQA1*05:05\DQB1*03:01 (DQ7) | 15.671140939597302 |
| DQA1*06:01\DQB1*03:01 (DQ7) | 8.89380530973451 |
| DQA1*01:01\DQB1*03:02 (DQ8) | 17.66242038216557 |
| DQA1*02:01 DQB1*03:02 (DQ8) | 15.149606299212607 |
| DQA1*03:01\DQB1*03:02 (DQ8) | 19.3809523809524 |
| DQA1*03:02\DQB1*03:02 (DQ8) | 23.880733944954077 |
| DQA1*02:01\DQB1*03:03 (DQ9) | 24.45871559633028 |
| DQA1*03:01\DQB1*03:03 (DQ9) | 16.539823008849613 |
| DQA1*03:02\DQB1*03:03 (DQ9) | 6.64957264957265 |
Peptide
β chain
55R
52P
α chain
