# Supplementary material for: HLA Epitopes: The Targets of Monoclonal and Alloantibodies Defined
Source: J Immunol Res. 2017 May 24;2017:3406230. doi: 10.1155/2017/3406230 (PMC5463109; doi:10.1155/2017/3406230)
Supplement: Supplementary file 11 [file 3406230.f11.pptx]

## Slide 1
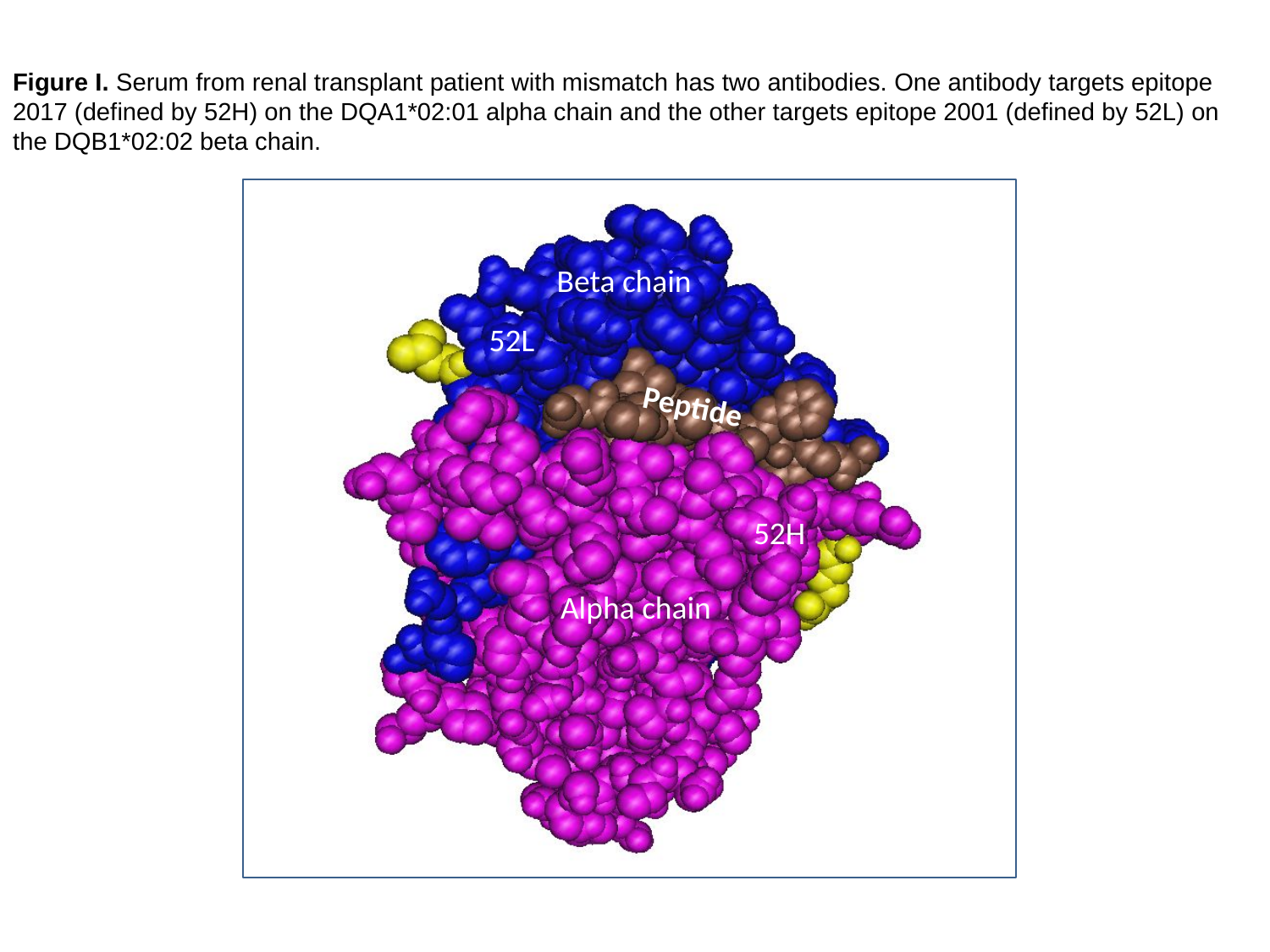

Figure I. Serum from renal transplant patient with mismatch has two antibodies. One antibody targets epitope 2017 (defined by 52H) on the DQA1*02:01 alpha chain and the other targets epitope 2001 (defined by 52L) on the DQB1*02:02 beta chain.
Beta chain
52L
Peptide
52H
Alpha chain
