# Supplementary material for: HLA Epitopes: The Targets of Monoclonal and Alloantibodies Defined
Source: J Immunol Res. 2017 May 24;2017:3406230. doi: 10.1155/2017/3406230 (PMC5463109; doi:10.1155/2017/3406230)
Supplement: Supplementary file 13 [file 3406230.f13.pptx]

## Slide 1
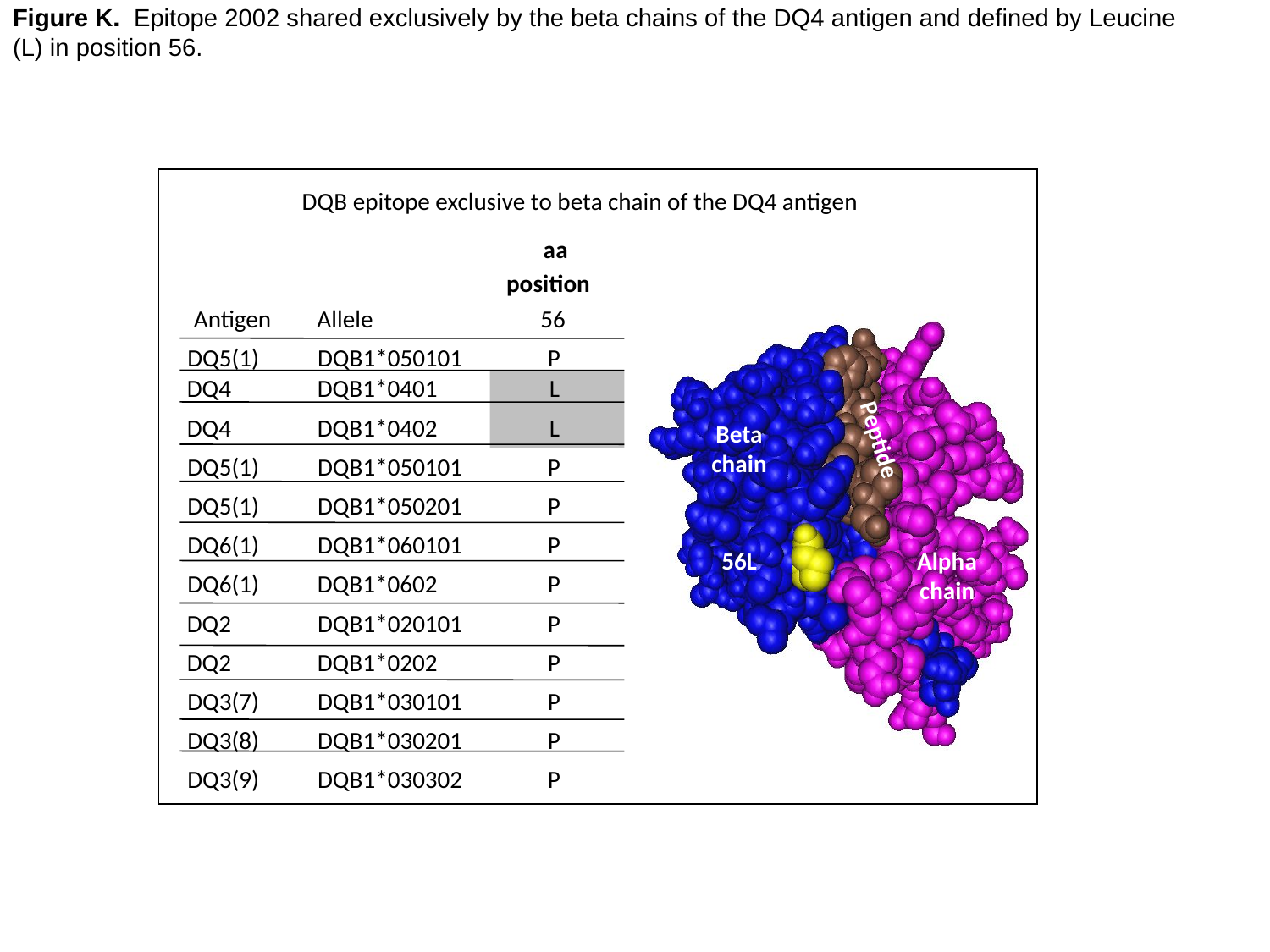

Figure K. Epitope 2002 shared exclusively by the beta chains of the DQ4 antigen and defined by Leucine (L) in position 56.
DQB epitope exclusive to beta chain of the DQ4 antigen
aa
position
Beta
chain
Peptide
56L
Alpha
chain
Antigen
Allele
56
DQ5(1)
DQB1*050101
P
DQ4
DQB1*0401
L
DQ4
DQB1*0402
L
DQ5(1)
DQB1*050101
P
DQ5(1)
DQB1*050201
P
DQ6(1)
DQB1*060101
P
DQ6(1)
DQB1*0602
P
DQ2
DQB1*020101
P
DQ2
DQB1*0202
P
DQ3(7)
DQB1*030101
P
DQ3(8)
DQB1*030201
P
DQ3(9)
DQB1*030302
P
