# Supplementary material for: HLA Epitopes: The Targets of Monoclonal and Alloantibodies Defined
Source: J Immunol Res. 2017 May 24;2017:3406230. doi: 10.1155/2017/3406230 (PMC5463109; doi:10.1155/2017/3406230)
Supplement: Supplementary file 14 [file 3406230.f14.pptx]

## Slide 1
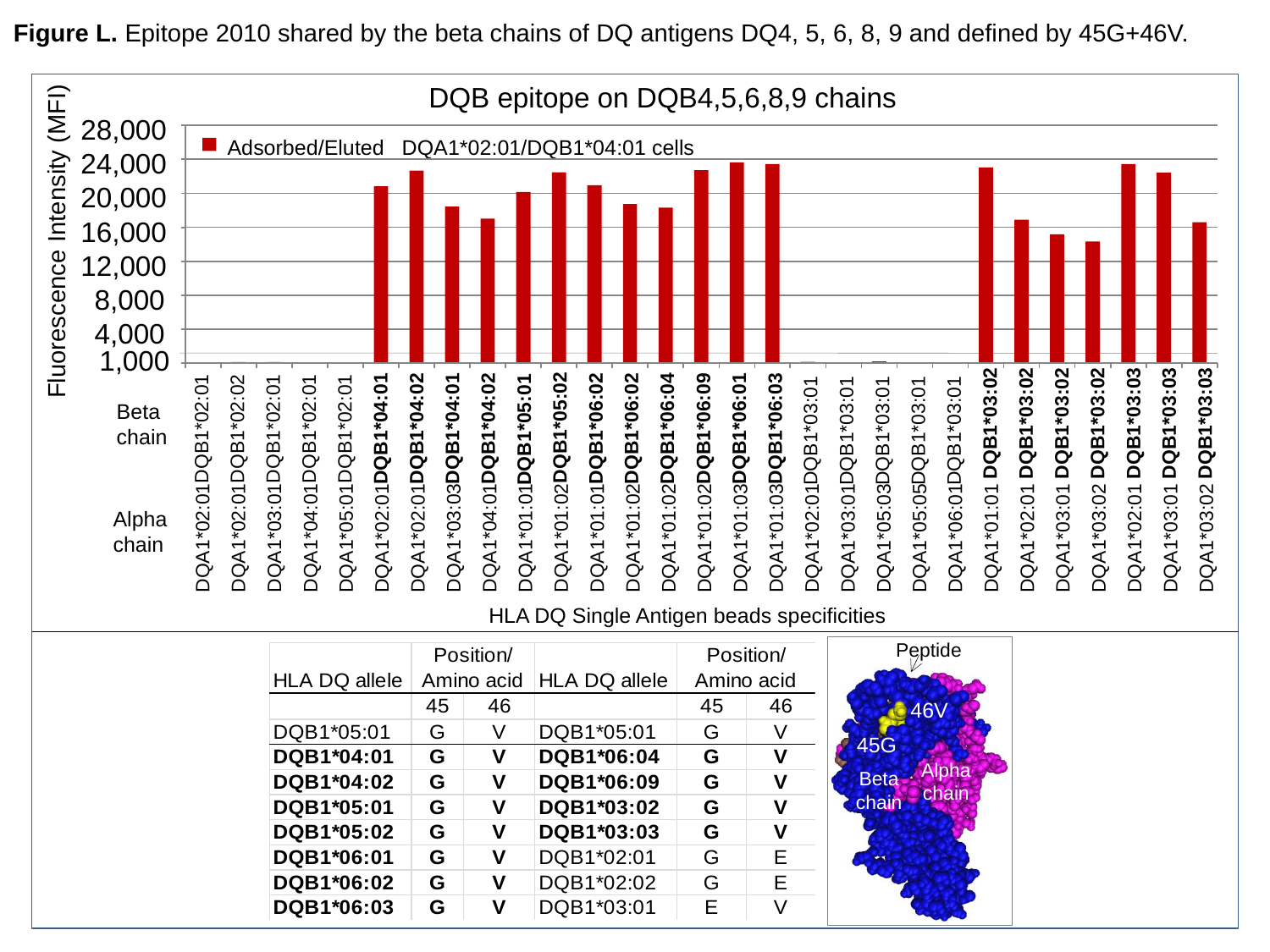

Figure L. Epitope 2010 shared by the beta chains of DQ antigens DQ4, 5, 6, 8, 9 and defined by 45G+46V.
DQB epitope on DQB4,5,6,8,9 chains
 28,000
Adsorbed/Eluted DQA1*02:01/DQB1*04:01 cells
 24,000
 20,000
 16,000
Fluorescence Intensity (MFI)
 12,000
 8,000
 4,000
 1,000
DQB1*03:02
DQB1*03:02
DQB1*03:02
DQB1*03:02
DQB1*03:03
DQB1*03:03
DQB1*03:03
DQB1*05:02
DQB1*04:02
DQB1*04:01
DQB1*04:02
DQB1*06:02
DQB1*06:02
DQB1*06:04
DQB1*06:09
DQB1*06:01
DQB1*06:03
DQB1*02:02
DQB1*02:01
DQB1*02:01
DQB1*02:01
DQB1*02:01
DQB1*04:01
DQB1*05:01
DQB1*03:01
DQB1*03:01
DQB1*03:01
DQB1*03:01
DQB1*03:01
Beta
chain
DQA1*03:03
DQA1*04:01
DQA1*01:01
DQA1*01:02
DQA1*01:01
DQA1*01:02
DQA1*02:01
DQA1*03:01
DQA1*04:01
DQA1*02:01
DQA1*01:02
DQA1*01:02
DQA1*01:03
DQA1*01:03
DQA1*02:01
DQA1*03:01
DQA1*05:03
DQA1*05:05
DQA1*06:01
DQA1*01:01
DQA1*02:01
DQA1*03:01
DQA1*03:02
DQA1*02:01
DQA1*03:01
DQA1*03:02
DQA1*05:01
DQA1*02:01
DQA1*02:01
Alpha
chain
HLA DQ Single Antigen beads specificities
Peptide
46V
45G
Alpha
chain
Beta
chain
