# Supplementary material for: HLA Epitopes: The Targets of Monoclonal and Alloantibodies Defined
Source: J Immunol Res. 2017 May 24;2017:3406230. doi: 10.1155/2017/3406230 (PMC5463109; doi:10.1155/2017/3406230)
Supplement: Supplementary file 15 [file 3406230.f15.pptx]

## Slide 1
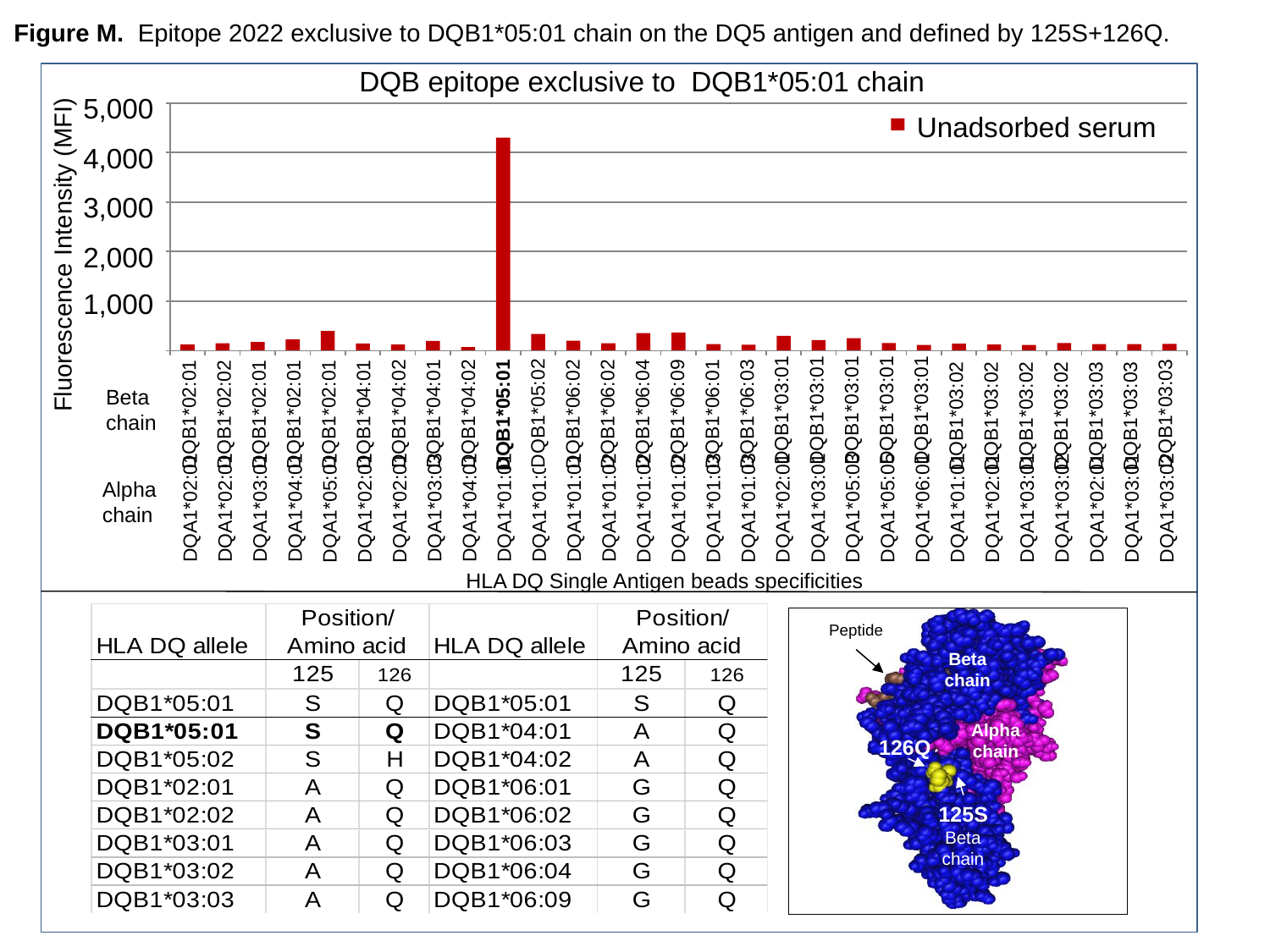

Figure M. Epitope 2022 exclusive to DQB1*05:01 chain on the DQ5 antigen and defined by 125S+126Q.
DQB epitope exclusive to DQB1*05:01 chain
 5,000
Unadsorbed serum
 4,000
 3,000
Fluorescence Intensity (MFI)
 2,000
 1,000
DQB1*03:01
DQB1*03:01
DQB1*03:01
DQB1*03:01
DQB1*03:01
DQB1*05:02
DQB1*03:03
DQB1*04:02
DQB1*04:01
DQB1*04:02
DQB1*06:02
DQB1*06:02
DQB1*06:04
DQB1*06:09
DQB1*06:01
DQB1*06:03
DQB1*02:02
DQB1*02:01
DQB1*02:01
DQB1*02:01
DQB1*02:01
DQB1*04:01
DQB1*05:01
DQB1*03:02
DQB1*03:02
DQB1*03:02
DQB1*03:02
DQB1*03:03
DQB1*03:03
Beta
chain
DQA1*03:03
DQA1*04:01
DQA1*01:01
DQA1*01:02
DQA1*01:01
DQA1*01:02
DQA1*02:01
DQA1*03:01
DQA1*04:01
DQA1*02:01
DQA1*01:02
DQA1*01:02
DQA1*01:03
DQA1*01:03
DQA1*02:01
DQA1*03:01
DQA1*05:03
DQA1*05:05
DQA1*06:01
DQA1*01:01
DQA1*02:01
DQA1*03:01
DQA1*03:02
DQA1*02:01
DQA1*03:01
DQA1*03:02
DQA1*05:01
DQA1*02:01
DQA1*02:01
Alpha
chain
HLA DQ Single Antigen beads specificities
Peptide
Beta
chain
Alpha
chain
126Q
125S
Beta
chain
