# Supplementary material for: HLA Epitopes: The Targets of Monoclonal and Alloantibodies Defined
Source: J Immunol Res. 2017 May 24;2017:3406230. doi: 10.1155/2017/3406230 (PMC5463109; doi:10.1155/2017/3406230)
Supplement: Supplementary file 16 [file 3406230.f16.pptx]

## Slide 1
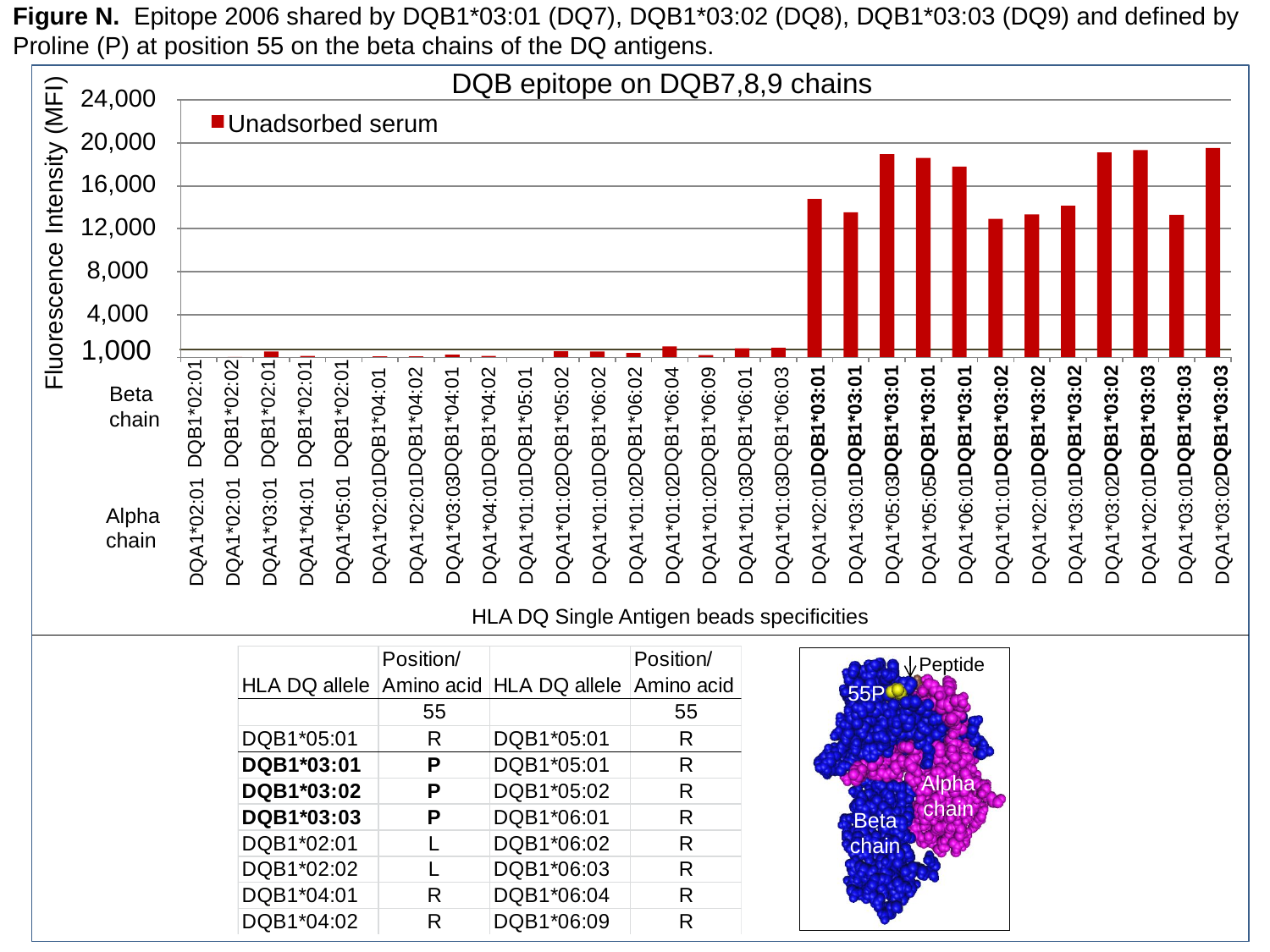

Figure N. Epitope 2006 shared by DQB1*03:01 (DQ7), DQB1*03:02 (DQ8), DQB1*03:03 (DQ9) and defined by Proline (P) at position 55 on the beta chains of the DQ antigens.
DQB epitope on DQB7,8,9 chains
 24,000
 20,000
 16,000
 12,000
 8,000
 4,000
 1,000
Unadsorbed serum
Fluorescence Intensity (MFI)
DQB1*02:02
DQB1*02:01
DQB1*02:01
DQB1*02:01
DQB1*02:01
DQB1*03:01
DQB1*03:01
DQB1*03:01
DQB1*03:01
DQB1*03:01
DQB1*03:02
DQB1*03:02
DQB1*03:02
DQB1*03:02
DQB1*03:03
DQB1*03:03
DQB1*03:03
DQB1*04:02
DQB1*04:01
DQB1*04:02
DQB1*05:02
DQB1*06:02
DQB1*06:02
DQB1*06:04
DQB1*06:09
DQB1*06:01
DQB1*06:03
DQB1*05:01
DQB1*04:01
Beta
chain
DQA1*03:03
DQA1*04:01
DQA1*01:01
DQA1*01:02
DQA1*01:01
DQA1*01:02
DQA1*01:02
DQA1*01:02
DQA1*01:03
DQA1*01:03
DQA1*02:01
DQA1*03:01
DQA1*05:03
DQA1*05:05
DQA1*06:01
DQA1*01:01
DQA1*02:01
DQA1*03:01
DQA1*03:02
DQA1*02:01
DQA1*03:01
DQA1*03:02
DQA1*05:01
DQA1*02:01
DQA1*02:01
DQA1*02:01
DQA1*03:01
DQA1*04:01
DQA1*02:01
Alpha
chain
HLA DQ Single Antigen beads specificities
Peptide
55P
Alpha
chain
Beta
chain
